# Viral and eukaryotic drivers of prokaryotic and antibiotic resistance gene diversity in wastewater microbiomes

**DOI:** 10.1186/s40168-025-02307-3

**Published:** 2026-01-10

**Authors:** Antonia Weiss, Alan Xavier Elena, Uli Klümper, Kenneth Dumack

**Affiliations:** 1 University of Cologne, Terrestrial Ecology, Institute of Zoology, Zülpicher Str. 47B, Köln, 50674 Germany; 2 Technische Universität Dresden, Institute of Hydrobiology, Zellescher Weg 40, Dresden, 01217 Germany; 3 University of Koblenz, Aquatic Ecosystem Analyses, Institute for Integrated Natural Sciences, Universitätsstraße 1, Koblenz, 56070 Germany

**Keywords:** Antibiotic resistance, Bacterial communities, Biotic interactions, Phages, Virus, Protists

## Abstract

**Background:**

Antibiotic resistance genes (ARGs) are proliferating in wastewater microbiomes, yet the biotic forces shaping their diversity remain poorly understood. Here, we integrate 14 months of metagenomic and metatranscriptomic data from a wastewater treatment plant to reveal that viruses and microeukaryotes, long-overlooked trophic actors, may play an important role in shaping bacterial and ARG diversity.

**Results:**

We show that viral and microeukaryotic communities exhibit strong seasonal dynamics that cascade through the microbial food web, significantly structuring prokaryotic communities and subsequently ARG profiles. Crucially, we find that viral and microeukaryotic diversity are positively associated with bacterial diversity, which in turn shapes ARG diversity, underscoring the regulatory potential of ecological interactions.

**Conclusions:**

Our findings challenge the abiotic-centric paradigm and establish the central role of multi-trophic interactions in shaping ARG dynamics in wastewater ecosystems.

Video Abstract

**Supplementary Information:**

The online version contains supplementary material available at 10.1186/s40168-025-02307-3.

## Background

Antimicrobial resistance is a major global public health concern and is thought to be driven mainly by the overuse of antibiotics in humans and animals worldwide, leading to a growing risk of untreatable infections [1]. Antibiotic resistance genes (ARGs) are recognized to occur within environmental bacteria [2] and have been detected across a wide range of environments [3–6], including activated sludge in wastewater treatment plant (WWTP) ecosystems [7]. Since WWTPs receive sewage from various origins, including hospitals, pharmaceutical industries, human households, or livestock treatment, they have been identified as important reservoirs for antibiotic resistant bacteria (ARB) and their associated ARGs [7, 8]. The presence and diversity of ARGs depend on the composition and diversity of the bacterial community in which they are encoded. Therefore, understanding the proliferation, diversity, and variation of ARGs requires identifying the factors that shape bacterial community composition in order to mitigate the associated risks to human health.

Bacterial communities in WWTPs are influenced by physicochemical factors, such as pH [9–11], as well as by biotic interactions. Fundamental ecological research highlights the crucial role of diversity in ecosystem functionality [12–14] and stability [15, 16]. In wastewater environments, heightened microbial diversity enhances the system's resilience and resistance to toxins [17]. The primary predators in wastewater are microeukaryotic protists, as well as microscopic metazoa [18–20]. Within WWTPs, predation has been observed to impact the microbial community composition and biomass [21–23]. Recent evidence highlights the role of predation in promoting prokaryotic diversity within wastewater; prokaryotic taxa richness, evenness, genetic diversity, and beta diversity all respond positively to high predation pressure [24].

Despite initial insights into mechanisms such as microeukaryotic predation, even less is known about the potential impact of viruses on shaping prokaryotic communities in WWTPs, although viruses have been suggested to function as efficient biocontrols of specific bacteria [25, 26]. Viruses represent a major factor contributing to bacterial mortality [27, 28]. Bacteriophages, viruses infecting prokaryotes, are considered the most abundant biological entities on Earth [29]. By causing bacterial lysis, facilitating horizontal gene transfer (HGT), and contributing to biochemical cycling, they are recognized as important drivers of bacterial abundance and community composition, as well as ecosystem functioning [30–33]. In marine environments, for instance, viral lysis is estimated to remove approximately 20% of the prokaryotic biomass daily, highlighting the strong regulatory role of viruses on microbial communities [34, 35]. Although viral community dynamics and virus-host interactions in WWTPs remain poorly understood, recent studies have highlighted their potential influence on microbial biomass, community dynamics, and WWTP system functioning [36–39]. Supporting this, Liu et al. [40] observed negative correlations between bacterial abundance and associated bacteriophages, suggesting that viruses may act as an important shaping factor of bacterial communities. In addition to their role in microbial community assembly, bacteriophages are also recognized as contributors to the HGT of ARGs [8, 38, 41]. There are two ways in which viruses can facilitate HGT: transduction and transformation. During transduction, ARGs can be packaged from an infected bacterial cell by a bacteriophage and subsequently transferred to other bacteria [42]. Transformation, in turn, can be facilitated by bacteriophage induced cell lysis. This involves the release of DNA containing ARGs into the environment, where it can be incorporated by other bacteria [43].

Taken together, these findings underline the importance of considering the wastewater microbiome as a whole, including all relevant trophic levels, to better understand the processes shaping microbial community composition and system functioning. Despite growing evidence of viral influences on microbial dynamics in WWTPs, studies that integrate viruses, prokaryotes, eukaryotes, and ARGs within a single framework remain rare. With this study, we aimed to address this gap by analyzing a publicly available time-series dataset from Herold et al. [10], which includes metagenomic and metatranscriptomic data from a WWTP microbiome. We hypothesize that viral infection and protistan predation play important roles in structuring prokaryotic and ARG diversity in wastewater microbiomes, perhaps with an even stronger impact than previously recognized abiotic drivers.

## Material and methods

For this study, we used publicly available data, comprising a well characterized time-series of microbial communities at the Schifflange municipal WWTP in Luxembourg (49°30′48.29"N; 6°1′4.53"E). The WWTP operates an activated sludge process with two aeration basins, each divided into an outer aerobic nitrification zone and an inner anaerobic denitrification zone. Samples were collected weekly using a levy cane to sample oleaginous biomass consisting of floating sludge islets from the surface of the anoxic tank (denitrification zone) within the aeration basin. In total, 53 samples were obtained over a period of 14 months. In a sequential co-isolation procedure, DNA and RNA were extracted and metagenomic as well as metatranscriptomic libraries were prepared for 100 bp-length paired-end Illumina sequencing. For a detailed description see Herold et al. [10]. Metagenomic and metatranscriptomic sequencing allow for an unbiased and comprehensive assessment of the entire microbial community, including prokaryotes, viruses, and protists. While the prokaryotic fraction of the data was analyzed in Herold et al. [10], and eukaryotes were analyzed in Heck et al. [44], we now additionally assessed the viruses and ARGs of this dataset and provide a comprehensive analysis.

### Data processing

The metagenomic data were used to assess non-living entities, i.e., DNA bacteriophages, of which double-stranded and tailed DNA bacteriophages comprise the majority to date [45], and ARGs. In contrast, the metatranscriptomic data were used to obtain the living fraction of the microbial community, including prokaryotes and eukaryotes. This approach provides a representative picture of the microbial community, particularly for eukaryotes, by avoiding biases related to PCR amplification, primer specificity, variation in ribosomal operon copy number, genome size or sequencing dead matter [46–50].

Raw metagenomic data were obtained from the NCBI Sequence Read Archive (SRA) under project accession number PRJNA230567. In total, 53 SRA files were downloaded. Two samples (2010–10–24, 2011–01–25) were excluded from further analyses due to a notably lower sequencing depth and their temporal distance from the main sampling phase. A complete overview of the included 51 samples is provided in Supplementary Table S1. Quality control of metagenomic reads was performed using FastQC v. 0.11.9 [51], and read trimming and filtering were conducted with TrimGalore v. 0.6.7 [52]. Adapter sequences were removed, reads with a quality score (Phred score) of < 30 were excluded, and the last five bases at the 3’ end of each read were cut. Taxonomic assignment of viral reads was carried out with Kraken2 v. 2.1.4 [53] against the NCBI RefSeq viral database [54] as reference, using the default confidence score of 0. An overview of viral reads per sample identified by Kraken2 is given in Supplementary Table S2. Viral reads were aggregated at the family level, forming operational taxonomic units (OTU) for subsequent analyses. Prokaryotic and eukaryotic community data were obtained from the previously processed metatranscriptomic dataset as described by Heck et al. (2023) [13]. Briefly, sequencing reads had been quality-filtered, assembled into contigs, and taxonomically assigned using BLASTN searches against the PR2 v. 4.11.1 database [55] for eukaryotic taxa and the SILVA 138 SSU Ref Nr. 99 database [56] for prokaryotic taxa. For further details, see Heck et al. (2023) [13]. Sequences assigned to chloroplasts, macroscopic metazoa, and Embryophyta were excluded. Taxa were binned at the genus level and later grouped by order to facilitate visualization. Reads that could not be assigned to the genus level were named after the last assigned taxonomic level (e.g., “family X”). Low-abundance OTUs, defined as singletons to quintuplets, were removed for both datasets. To improve dataset comparability, three samples identified as outliers (2011–04–05, 2011–03–29, 2011–03–21) were excluded. Rarefaction curves were generated for viral sequences (Supplementary Figure S1), and prokaryotic and eukaryotic sequences combined (Supplementary Figure S2) to assess sequencing depth and evaluate whether the diversity within each sample was captured. To ensure comparability between samples, the viral dataset was rarefied to 486 viral reads per sample, corresponding to the lowest number of viral sequences observed across all samples. Prokaryotic and eukaryotic sequences were rarefied to a depth of 1,066,681 reads per sample, which led to the exclusion of three samples (2011–11–23, 2011–11–16, 2011–11–29). All further analyses were therefore conducted on the final set of 45 samples for both datasets.

### ARG quantification and normalization

ARGs were quantified using a hidden Markov model (HMM)-based pipeline and the Structured Antibiotic Resistance Gene (SARG) database as described by Yang et al. [57] and Yin et al. [58]. In brief, the metagenomic reads were mapped to the SARG reference database and, due to the varying length of ARG genes, normalized based on gene length to avoid overrepresentation of shorter reads. ARG abundances were then normalized to 16S rRNA gene abundances, which were processed in parallel using the same HMM approach. Individual ARGs were grouped according to the antibiotic classes they confer resistance to. All subsequent analyses involving ARGs were based on these normalization values, reflecting ARG abundance per 16S rRNA gene copy rather than absolute read counts.

### Statistical analyses

All statistical analyses and graph visualizations were performed with R v. 4.4.2 [59] using RStudio and the following packages: ape v. 5.8.1 [60], car v. 3.1.3 [61], dplyr v. 1.1.4 [62], FSA v. 0.10.0. [63], ggplot2 v. 3.5.1 [64], ggrepel v. 0.9.6 [65], lattice v. 0.22.6 [66], lavaan v. 0.6.19 [67], lubridate v. 1.9.4 [68], tibble v. 3.2.1 [69], tidyr v. 1.3.1 [70], tidySEM v. 0.2.8 [71], tidyverse v. 2.0.0 [72], readr v. 2.1.5 [73], reshape2 v. 1.4.4 [74], scales v. 1.3.0 [75], svglite v. 2.1.3 [76], vegan v. 2.6.8 [77]. Graph arrangements were conducted using Inkscape v. 1.3.2 [78].

To assess the proportion of viral, prokaryotic, and eukaryotic reads, the relative abundance of each group was calculated per sample based on the total number of raw reads, and visualized using violin plots. An overview of the total number of raw reads from metagenomic and metatranscriptomic sequencing is given in Supplementary Table S3. For all violin plots, samples were grouped by season to examine potential temporal patterns in relative read proportions. Seasons were defined as follows: spring (March–May), summer (June–August), autumn (September–November), and winter (December-February). Differences in relative sequence abundance over seasons were tested using the non-parametric Kruskal–Wallis rank sum test [79], as the assumption of normality was not met, as tested with the Shapiro–Wilk test [80]. For post hoc analysis, Dunn’s test [81] with Benjamini–Hochberg correction [82] was conducted to identify seasonal differences. To visualize changes in community composition over time, stacked bar plots were generated from rarefied count tables. Visualization focused on the ten most abundant viral taxa, and the fifteen most abundant prokaryotic and eukaryotic taxa, based on total read counts summed across all samples. For ARGs, the ten most abundant resistance classes were selected based on the sum of normalized abundances relative to 16S rRNA gene sequence counts across all samples. In addition, the relative abundance of individual viral, prokaryotic, and eukaryotic taxa as well as ARG classes, were statistically tested to assess seasonal variation.

To investigate microbial turnover over time, time-decay analyses [83] were performed to assess whether the microbial community structure and the resistome exhibited a decay pattern with increasing temporal distance between the sampling dates. Bray–Curtis dissimilarities were calculated between all pairwise sample comparisons. Similarities were obtained as (1 - Bray–Curtis dissimilarity) and plotted against the temporal distance between samples. The relationship between community similarity and time difference was assessed using linear regression (Supplementary Figure S3).

Non-metric multidimensional scaling (NMDS) based on Bray–Curtis dissimilarities was used to assess beta diversity of viral, prokaryotic, eukaryotic community structures, and the resistome, over time in further detail. Ordinations were performed in two dimensions (k = 2) using the metaMDS() function from the vegan package. The resulting stress values were 0.05 for viruses, 0.079 for prokaryotes, 0.124 for eukaryotes, and 0.058 for ARGs. To identify potential drivers of community variation, environmental variables (water temperature and pH; as described in Herold et al., [10]) as well as the ten most abundant viral taxa were fitted as vectors using the envfit() function. An overview of environmental parameters measured for all samples used in this study is given in Supplementary Table S4. Only statistically significant vectors (*p* < 0.05) were displayed in the ordination plots.

Principal coordinate analysis (PCoA) was used as a metric measure of community structure across viral, prokaryotic, eukaryotic, and ARG communities. Ordinations were calculated using the cmdscale() function based on Bray–Curtis dissimilarities, with distance matrices generated with the vegdist() function from the vegan package. For each community, the first two principal coordinate axes (PC1 and PC2) were retained. The proportion of variance explained by each axis was 49.79% (PC1) and 32.87% (PC2) for viruses, 41.54% and 17.84% for eukaryotes, 67.24% and 12.86% for prokaryotes, and 86.49% and 3.97% for ARGs. Supplementary Figure S4 provides an overview of all PCoA results. PC1 was used for further analyses as a representative metric of community structure. To evaluate the influence of environmental and biotic predictors on beta diversity, permutational multivariate analysis of variance (PERMANOVA) was conducted using the adonis2() function from the vegan package. Analyses were performed with 999 permutations and Bray–Curtis distance as dissimilarity matrix. Explanatory variables included season, water temperature, pH, and the PC1 of each respective community dataset. The variables were ordered hierarchically (by = “terms”) to assess their individual contribution to community variation. PERMANOVA was performed separately for each community. An overview is provided in Supplementary Figure S5.

To investigate specific associations between viral and microbial taxa as well as ARG classes, pairwise Spearman’s rank correlations were assessed using relative abundance data. The analysis included the ten most abundant viral families, the fifteen most abundant prokaryotic orders, and the ten most abundant ARG classes, selected based on total cumulative abundance across all samples. Additional correlations between the ten most abundant viral families and the fifty most abundant prokaryotic genera are provided in Supplementary Figure S6. Due to the non-normal distribution of the relative abundance data, confirmed by the Shapiro–Wilk test, Spearman’s rank correlation was applied instead of Pearson correlation, using the cor.test() function [84]. The resulting correlation matrices were visualized as heatmaps. Spearman correlation coefficients for correlations between viral taxa and ARG classes, as well as between viral and prokaryotic taxa, are shown in Supplementary Tables S5, S6 and S7 respectively.

Structural equation modeling was used to test directed associations between seasonality and viral, prokaryotic, eukaryotic, and ARG diversity. SEM allowed us to test multiple interaction pathways and was therefore well suited to investigate the complex multi-trophic associations within the WWTP system [85]. Biodiversity metrics were calculated as Shannon–Wiener diversity indices from rarefied count data and used as community-scale biotic variables. The categorical variable season was numerically encoded to allow its inclusion as a predictor. SEM was implemented using the lavaan package, and the resulting unstandardized path coefficients (estimate) were visualized with the tidySEM package. Model fit indices of the SEM are displayed in Supplementary Table S8, and the path coefficients for each tested relationship are shown in Supplementary Table S9.

## Results

Across all 45 samples, the detected ARGs from the metagenomic dataset were grouped into 21 resistance classes based on the antibiotic classes they confer resistance to. From the same dataset, approximately 22,000 viral reads were identified and assigned to 32 different viral families. For prokaryotic and eukaryotic taxa from the metatranscriptomic dataset, a combined total of approximately 47.9 million SSU rRNA gene sequences were obtained, which were assigned to 1,255 prokaryotic and 3,088 eukaryotic genera, respectively.

### Seasonal dynamics of relative abundance and alpha diversity of ARGs, viruses, prokaryotes, and eukaryotes

**Fig. 1:**
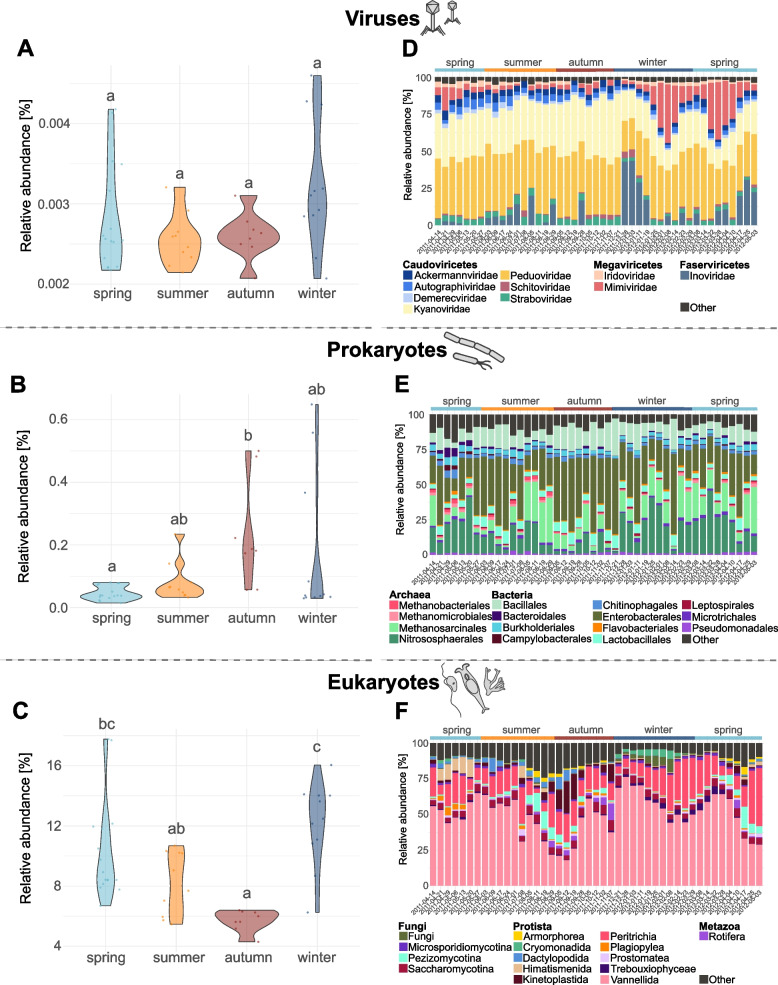
Seasonal abundance dynamics of viruses, prokaryotes, and eukaryotes and community compositions. **A**-**C** Violin plots showing seasonal distributions of relative read abundances for (**A**) viruses, (**B**) prokaryotes, and (**C**) eukaryotes. Each dot represents an individual sample, grouped by season: spring (light blue), summer (yellow), autumn (red), and winter (dark blue). Y-axis scaling differs between the figures due to differences in overall abundance levels among the datasets. Different lowercase letters (**a**-**c**) above the violins indicate statistically significant differences between seasons (*p* < 0.05), based on Dunn’s post hoc test with Benjamini–ochberg correction. **D**-**F** Stacked bar plots showing the community composition across all samples based on relative abundances of the (**D**) ten most abundant viral families, (**E**) fifteen most abundant prokaryotic orders, and (**F**) fifteen most abundant eukaryotic orders. Each bar represents one sample, ordered chronologically by sampling date (x-axis). Taxa are color-coded as indicated in the legends. All further taxa, i.e. below the threshold of the ten most abundant viral families and fifteen most abundant prokaryotic and eukaryotic orders, are cumulated to “Other”

**Fig. 2:**
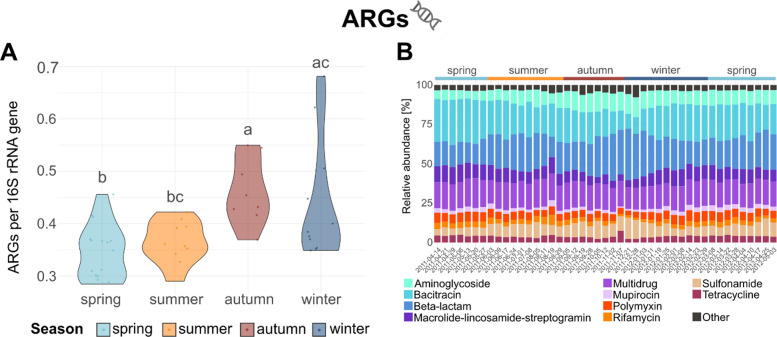
Seasonal abundance dynamics of antibiotic resistance gene (ARG) and the resistome. **A** Violin plot showing the seasonal distribution of ARG abundances normalized to 16S rRNA gene counts. Each dot represents an individual sample, grouped by season: spring (light blue), summer (orange), autumn (red), and winter (dark blue). Different lowercase letters (**a**-**c**) above the violins indicate statistically significant differences between seasons (*p* < 0.05), based on Dunn’s post hoc test with Benjamini–ochberg correction. **B** Stacked bar plot showing the relative abundance of the ten most abundant ARG classes across all samples. Samples are ordered chronologically by sampling date (x-axis). ARG classes are color-coded as indicated in the legends. All further ARG classes, i.e. below the threshold of the ten most abundant ARG classes, are cumulated to “Other” and include: antibacterial-fatty-acid, chloramphenicol, defensin, florfenicol, fosfomycin, novobiocin, pleuromutilin-tiamulin, quinolone, streptothricin, trimethoprim, and vancomycin

In general, normalized ARG relative abundances varied significantly across seasons (*p* = 0.001; Fig. 1A) with autumn differing from spring (*p* = 0.002) and summer (*p* = 0.016) but not from winter (*p* = 0.232) and winter differing from spring (*p* = 0.027) but not from summer (*p* = 0.153). Viral read counts remained relatively consistent across seasons (Fig. 2A), with no significant differences detected (*p* = 0.127). In contrast, prokaryotic and eukaryotic read abundances varied significantly across seasons (prokaryotes: *p* = 0.011; eukaryotes: *p* < 0.001; Fig. 2B, C). Prokaryotic read abundances increased during summer and significantly differed between autumn and spring (*p* = 0.006). Eukaryotic read abundances peaked in winter and showed significant differences between winter and autumn (*p* < 0.001), winter and summer (*p* = 0.013), and autumn and spring (*p* = 0.002).

Significant seasonal differences in ARG abundance were also observed when separately analyzing each of the antibiotic classes to which they confer resistance to, based on those ten antibiotic classes with the highest abundance of corresponding ARGs (Fig. 1B). For example, those ARGs associated with resistance to bacitracin (mean = 0.206, SD = 0.044), beta-lactams (mean = 0.202, SD = 0.048), or multiple drugs (mean = 0.162, SD = 0.011) exhibited the highest relative abundances throughout and also showed significant seasonal variations (bacitracin: *p* = 0.015; beta-lactams: *p* = 0.036; multiple drugs: *p* = 0.032). Bacitracin resistance genes were found to be highest in spring (mean = 0.233, SD = 0.039) and lowest in autumn (mean = 0.176, SD = 0.033). Beta-lactam resistance genes were observed to be highest in autumn (mean = 0.229, SD = 0.047) and lowest in spring (mean = 0.177, SD = 0.037) and multiple drug resistance peaked in summer (mean = 0.167, SD = 0.01) and was lowest in winter (mean = 0.152, SD = 0.011).

With respect to viral community composition, the ten most abundant families were dominated by *Peduoviridae* (mean = 0.371, SD = 0.065) and *Kyanoviridae* (mean = 0.27, SD = 0.075), both bacteriophage families belonging to the class *Caudoviricetes* (*Duplodnaviria*) (Fig. 2D). Other dominant bacteriophage families among the top ten viral taxa included *Autographiviridae* (mean = 0.039, SD = 0.014), *Straboviridae* (mean = 0.038, SD = 0.012), *Ackermannviridae* (mean = 0.037, SD = 0.012), *Demerecviridae* (mean = 0.021, SD = 0.009), and *Schitoviridae* (mean = 0.013, SD = 0.012), also from the class *Caudoviricetes*, as well as *Inoviridae* (mean = 0.076, SD = 0.113) from the class *Faserviricetes* (*Monodnaviria*). While the relative abundance of *Peduoviridae* remained stable over time (*p* = 0.37), others, such as *Kyanoviridae* and *Autographiviridae*, exhibited significant seasonal variation (*p* < 0.001 and* p* = 0.007, respectively). For *Kyanoviridae*, the highest mean relative abundance was observed in autumn (mean = 0.371, SD = 0.047) and the lowest in winter (mean = 0.232, SD = 0.074). For *Autographiviridae*, abundances peaked in summer (mean = 0.047, SD = 0.015) and were lowest in winter (mean = 0.027, SD = 0.009). Additionally, two viral families belonging to the class *Megaviricetes* (*Varidnaviria*) and known to infect eukaryotes contributed to the overall composition of the viral community, namely *Mimiviridae* (mean = 0.088, SD = 0.105), which infect amoebae, and *Iridoviridae* (mean = 0.02, SD = 0.012), which infect both vertebrates and invertebrates.

The prokaryotic community composition was largely composed of members of the bacterial orders *Enterobacterales* (*Gammaproteobacteria*) (mean = 0.308, SD = 0.14), *Bacillales* (mean = 0.118, SD = 0.056) and *Lactobacillales* (mean = 0.052, SD = 0.02) (*Bacilli*), as well as members of the archaeal orders *Nitrososphaerales* (*Nitrososphaeria*) (mean = 0.163, SD = 0.111) and *Methanosarcinales* (*Methanomicrobia*) (mean = 0.124, SD = 0.097) (Fig. 2E). Several taxa, such as *Bacillales* (*p* = 0.013), *Enterobacterales* (*p* = 0.027), and *Lactobacillales* (*p* = 0.021), exhibited significant seasonal variations in their relative abundances. *Bacillales* mean relative abundance was highest in autumn (mean = 0.168, SD = 0.038) and lowest in spring (mean = 0.091, SD = 0.039). *Enterobacterales* mean relative abundance peaked in autumn as well (mean = 0.431, SD = 0.104) and was lowest in spring (mean = 0.246, SD = 0.112). The same pattern was also observed for the mean relative abundance of *Lactobacillales*, peaking in autumn (mean = 0.07, SD = 0.016) and was lowest in spring (mean = 0.043, SD = 0.016). In contrast, taxa such as *Methanosarcinales* (*p* = 0.86) and *Pseudomonadales* (*p* = 0.607) showed no significant seasonal differences.

Within the eukaryotic community, the most abundant taxa, including *Vannellida* (*Discosea*) (mean = 0.497, SD = 0.143) and *Peritrichia* (*Oligohymenophorea*) (mean = 0.16, SD = 0.087), maintained relatively stable abundances across seasons, with no significant differences detected (*Vannellida*: *p* = 0.059 and *Peritrichia*: *p* = 0.919; Fig. 2F). In contrast, other taxa such as *Kinetoplastida* (*Kinetoplastea*), *Cryomonadida* (*Thecofilosea*), and *Dactylopodida* (*Discosea*), showed seasonal variation in relative abundances (each with *p* < 0.001). The mean relative abundance for *Kinetoplastida* was highest in autumn (mean = 0.099, SD = 0.078) and lowest in winter (mean = 0.008, SD = 0.004). The opposite was found for *Cryomonadida*, for which the highest mean relative abundance was observed in winter (mean = 0.032, SD = 0.019) and the lowest in autumn (mean < 0.001, SD < 0.001). For *Dactylopodida*, the highest mean relative abundance was found in summer (mean = 0.032, SD = 0.025) and the lowest in winter (mean = 0.001, SD = 0.001). In addition to the previously mentioned microeukaryotes, which are phagocytic predators of bacteria, the fungal lineage *Saccharomycotina* (*Saccharomycetes*) (mean = 0.045, SD = 0.012) also maintained relatively stable abundances across seasons (*p* = 0.272).

### Beta diversity and seasonal patterns of ARGs, viruses, prokaryotes, and eukaryotes

Overall, the resistome as well as the viral, prokaryotic, and eukaryotic communities showed significant time-decay in community similarity (Supplementary Figure S3,* p* < 0.05), with negative slopes indicating decreasing similarity over increasing temporal distance.

**Fig. 3:**
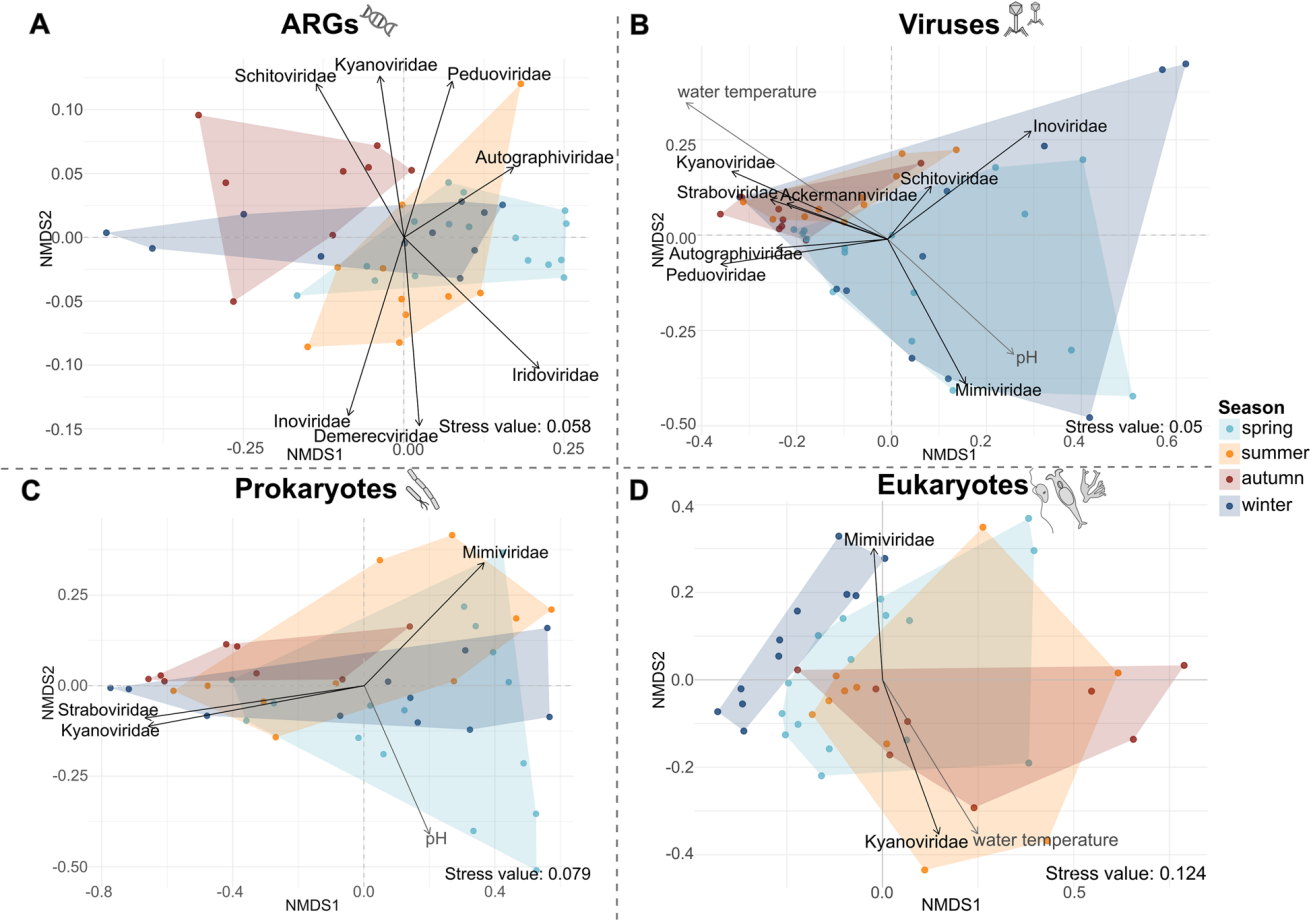
Influences on ARGs, viral, prokaryotic, and eukaryotic community composition. **A**-**D** Non-metric multidimensional scaling (NMDS) ordinations based on Bray–Curtis dissimilarities illustrating seasonal structuring of (**A**) ARGs, **B** viruses, **C** prokaryotes, and (**D**) eukaryotes. Each dot represents one sample, color-coded by season: spring (light blue), summer (yellow), autumn (red), and winter (dark blue). Polygons encompass samples from the same season. Significant environmental vectors (grey arrows; *p* < 0.05) indicate the direction and strength of correlation with community composition. **A** displays the ARG structure, with significant viral vectors. **B** shows the viral community structure, with the significant environmental vectors (grey) water temperature and pH, as well as significant viral taxa as vectors. **C** shows the prokaryotic community structure, with the significant environmental vector (grey) pH and the significant viral taxa as vectors. **D** shows the eukaryotic community structure, with the significant environmental vector (grey) water temperature and significant viral taxa as vectors

In further detail, the NMDS ordination of ARG beta diversity revealed distinct seasonal clustering with little overlap. Spring and summer samples clustered more closely in ordination space, while autumn samples were clearly separated (Fig. 3A). Measured environmental parameters showed no effect on the resistome. *Kyanoviridae* and *Schitoviridae* increased towards autumn samples, whereas *Inoviridae* and *Demerecviridae* increased with summer samples. *Peduoviridae*, *Autographiviridae*, and *Iridoviridae* increased with spring samples. Significant influences on ARG structure were detected for season (R^2^ = 27.7%, *p* = 0.001), viral community structure (R^2^ = 6.4%, *p* = 0.001), and prokaryotic community structure (R^2^ = 3.5%, *p* = 0.001).

Viral beta diversity analysis revealed a separation of samples along a seasonal gradient, with summer and autumn communities clustering closely, while spring and winter samples showed more variation (Fig. 3B). Higher water temperatures were associated with summer and autumn samples, while higher pH values were associated with winter and spring samples. In total, eight viral taxa showed significant associations with the ordination space, aligning with environmental gradients of water temperature and pH. *Kyanoviridae*, *Straboviridae*, and *Ackermannviridae* increased with water temperature and decreased with increasing pH. *Inoviridae* and *Mimiviridae* increased with higher pH and lower water temperatures. Season (R^2^ = 15.6%, *p* = 0.001), water temperature (R^2^ = 11.3%, *p* = 0.002), and pH (R^2^ = 5.8%, *p* = 0.026) showed significant influence on the viral community structure. Additional influences were observed from prokaryotic (R^2^ = 4.7%, *p* = 0.011) and eukaryotic (R^2^ = 4.3%, *p* = 0.029) community structures, and the resistome (R^2^ = 12.9%, *p* = 0.001).

NMDS based on prokaryotic beta diversity showed no clear separation of samples by season (Fig. 3C). Although slight seasonal tendencies were visible, the overall sample distribution showed substantial overlap across all seasons, indicating that seasonality had only a weak influence on the prokaryotic community structure (R^2^ = 8.4%, *p* = 0.215). *Mimiviridae*, *Straboviridae*, and *Kyanoviridae* showed correlations with the distribution of the prokaryotic community. *Kyanoviridae* and *Straboviridae* increased in association with winter samples, while *Mimiviridae* increased with pH and in association with summer samples. Even though the influence of the viral community structure on the prokaryotic community structure appeared relatively weak (R^2^ = 5.6%, *p* = 0.079), a visible trend validated further investigation using Spearman correlation and structural equation modeling (see next section). Nevertheless, significant effects were observed for the eukaryotic (R^2^ = 6.4%, *p* = 0.047) community structure and the resistome (R^2^ = 8.2%, *p* = 0.022).

The eukaryotic beta diversity displayed a seasonal separation of the samples with partially overlapping but distinguishable clusters (Fig. 3D). Summer and autumn samples tended to cluster closely together, while winter samples were more separated and grouped more closely with spring samples. Although seasonal clusters were not completely distinct, the spatial arrangement indicated a gradual seasonal shift in eukaryotic beta diversity, with season explaining a significant, and large, portion of the variation (R^2^ = 20.9%, *p* = 0.001). Water temperature was identified as an environmental factor shaping eukaryotic community structure, as indicated by the clustering of summer and autumn samples at higher temperature values. In addition, two viral taxa showed significant associations with the ordination of the eukaryotic community structure. *Mimiviridae* increased with lower water temperatures, while *Kyanoviridae* increased with warmer water temperatures. Further, a significant effect of the viral community structure on the eukaryotic community structure could be revealed (R^2^ = 7.0%, *p* = 0.001).

### Correlations and putative associations among ARGs, viruses, prokaryotes, and eukaryotes

**Fig. 4:**
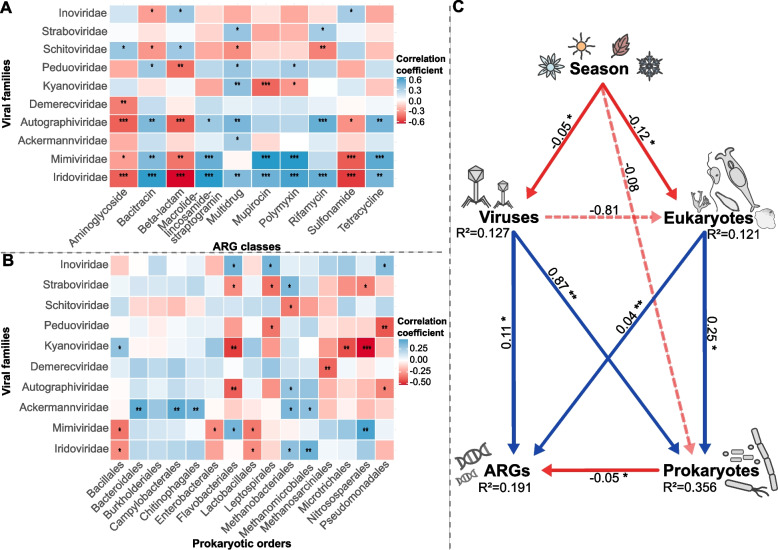
Correlations and associations between ARGs, viruses, prokaryotes, and eukaryotes. **A**-**B** Spearman correlation heatmaps showing associations between viral families and ARG classes as well as prokaryotic taxa. Correlation coefficients are color-coded (blue = positive, red = negative), and significance is indicated with asterisks *p ≤ 0.05, **p ≤ 0.01, ***p ≤ 0.001. **A** Correlations between the ten most abundant viral families and the ten most abundant ARG classes are shown. **B** Correlations between the ten most abundant viral families and fifteen most abundant prokaryotic orders are shown. **C** Structural equation model (SEM) illustrating directed associations between seasonality and the Shannon diversity of viral, prokaryotic, eukaryotic and ARG communities. Arrows indicate the direction of hypothesized relationships. Numbers next to the arrows represent estimate path coefficients, with significance levels denoted by asterisks (*p ≤ 0.05, **p ≤ 0.01, ***p ≤ 0.001). Blue arrows indicate positive associations and red arrows indicate negative associations; solid arrows represent significant paths; dashed arrows indicate non-significant paths. R^2^ values are noted below each variable

To further explore potential associations between viral families, ARG classes, and prokaryotic orders, pairwise Spearman correlations based on relative abundances were calculated. The resulting heatmaps revealed virus-specific correlation patterns. In the virus-ARG correlation matrix (Fig. 4A), both strong negative and strong positive correlations emerged, particularly for the viral families *Mimiviridae*, *Iridoviridae*, *Autographiviridae*, and *Peduoviridae*, which showed significant correlations with several ARG classes. For instance, *Autographiviridae* showed strong negative correlations with the ARG classes aminoglycoside and beta-lactam (correlation coefficients −0.495 and −0.503, respectively) and strong positive correlations with the ARG classes bacitracin and multiple drugs (correlation coefficients 0.430 and 0.45, respectively). *Peduoviridae* also exhibited strong negative correlations with beta-lactam (correlation coefficient −0.447) and strong positive correlations with bacitracin and multiple drugs (correlation coefficients 0.332 and 0.348, respectively). In contrast, families like *Kyanoviridae* and *Straboviridae* showed strong correlations with prokaryotic taxa, but their correlations with ARG classes were generally weaker. For example, *Kyanoviridae* and *Straboviridae* exhibited several negative correlations with different prokaryotic taxa, whereas families like *Ackermannviridae* and *Inoviridae* were positively associated with selected taxa (Fig. 4B). Further, strong negative correlations were identified for *Peduoviridae* and *Autographiviridae* with *Pseudomonadales* (correlation coefficients −0.418 and −0.319, respectively). For archaeal taxa from the *Euryarchaeota*, both positive and negative correlations with bacteriophage families were observed. For example, *Demerecviridae* showed negative correlations with *Methanosarcinales* (correlation coefficient −0.385), whereas both *Ackermannviridae* and *Autographiviridae* showed positive correlations with *Methanobacteriales* (correlation coefficients 0.361 and 0.294, respectively).

To add comprehensive context to the found correlations, we applied structural equation modeling, revealing several positive and negative associations (Fig. 4C). Seasonality showed a significant negative association (*p* = 0.01) with the alpha diversity of the viral community. Similarly, a significant negative association (*p* = 0.03) was found between seasonality and eukaryotic diversity. In contrast, seasonality showed no significant association with prokaryotic diversity. Viral diversity displayed significant positive association (*p* = 0.01) with prokaryotic diversity. Additionally, a significant positive association (*p* = 0.02) was observed between viral and ARG diversity. No significant association was found between viral and eukaryotic diversity. Eukaryotic diversity was positively associated (*p* = 0.02) with prokaryotic diversity. Prokaryotic diversity showed a significant negative association (*p* < 0.01) with ARG diversity. Overall, the model explained 35.6% of the variance in prokaryotic diversity, 19.1% of the variation in ARG diversity, 12.7% of the variation in viral diversity, and 12.1% of the variance in eukaryotic diversity.

## Discussion

Wastewater environments are known hotspots for ARGs, whose occurrence and diversity primarily depend on bacterial diversity. Understanding which factors shape prokaryotic diversity in these systems is therefore crucial. While numerous studies have examined correlations between ARGs and bacterial diversity, and the influence of abiotic factors on prokaryotic communities, the role of biotic influences, such as predation and infection pressure, remains poorly understood. In this study, we present a comprehensive analysis of these factors and their combined influence on prokaryotic diversity to understand ARG variation within the wastewater microbiome.

Among these biotic factors, we found a diverse viral community within the wastewater microbiome, dominated by members of the class *Caudoviricetes*. This class includes the majority of currently known bacteriophages and consists of tailed double-stranded DNA viruses [86]. Members of this class are dominant in many habitats, such as the deep sea [87], terrestrial environments [88], the human gut [89], and have already been found to dominate wastewater ecosystems [90, 91]. Within *Caudoviricetes*, we identified *Peduoviridae*, *Kyanoviridae*, and *Autographiviridae* as the most abundant families, all formerly grouped under *Myoviridae* and *Podoviridae* [92]. These families differ in their replication strategies. *Peduoviridae* are capable of switching between lysogenic and lytic cycles and are thus considered temperate bacteriophages, whereas *Kyanoviridae* exhibit an obligately lytic lifestyle [92, 93]. *Autographiviridae* have likewise been described as strictly lytic, although recent findings suggest that lysogeny may occur in some cases [94]. We observed seasonal variation within the viral community, with several viral families showing changes in relative abundance across seasons. For instance, *Kyanoviridae* and *Autographiviridae* showed significant seasonal variation in relative abundance, with *Kyanoviridae* being most abundant in autumn and *Autographiviridae* peaking in summer. This variation may be linked to environmental factors, as they appeared to influence the overall viral community structure, with water temperature and pH showing significant effects on viral beta diversity. These findings align with previous studies in marine systems showing that viral communities significantly varied with temperature [95]. In contrast, *Peduoviridae* exhibited no significant variation in relative abundance throughout the seasons, indicating a relatively stable occurrence across the sampling period. Similar patterns of temporal stability in viral communities have been observed in other aquatic ecosystems, where certain phage groups remain stable despite seasonal environmental fluctuations [96]. Ecological hypotheses such as the “kill-the-winner” and “piggyback-the-winner” hypotheses have been described to explain seasonal shifts in phage-host dynamics [30, 97, 98]. In our dataset, prokaryotic read abundances increased during summer, indicating higher microbial activity, which corresponded with exceptionally high abundances of lytic bacteriophages. The stable pattern of *Peduoviridae,* however, indicates that some viral taxa may persist throughout the year irrespective of these seasonal trends. Overall, viral read abundance remained relatively stable across the seasons, which may reflect a dynamic but balanced viral community.

We found significant correlations between viral beta diversity and both eukaryotic and prokaryotic community structures. According to previous findings, seasonal changes in water temperature affected the eukaryotic community composition, which in turn influenced the structure of the prokaryotic community [44]. This shift in prokaryotic composition may also affect the viral community, as viruses depend on host organisms for replication. A similar pattern was observed in marine ecosystems, where viral community structure was found to follow changes in host communities that were shaped by environmental conditions [95]. To investigate potential host associations in more detail, we examined viral and prokaryotic correlations. *Peduoviridae* showed significant negative correlations with *Pseudomonadales*, and negative but non-significant trends with *Flavobacteriales* and *Burkholderiales*, all of which are Gram-negative bacterial orders and known as potential host candidates [92]. Similarly, *Autographiviridae*, which are known to infect *Gammaproteobacteria* [94], were significantly negatively correlated with *Pseudomonadales*. Interestingly, *Enterobacterales* were among the most abundant bacterial taxa, but did not show correlations with either *Peduoviridae* or *Autographiviridae*, despite being potential host candidates. These results may reflect complex virus-host dynamics not fully captured by abundance correlations alone, given the high host specificity of many bacteriophages [99, 100].

While viruses influence bacterial diversity through infection, eukaryotes, particularly bacterivorous protists, may exert selective grazing pressure on the bacterial community. In our dataset, *Vannellida* were identified as the most abundant taxon and likely played a central role in bacterial regulation. Sessile ciliates of the *Peritrichia*, such as *Vorticella*, were also present across all samples and are known to contribute to biofilm formation, facilitate nutrient cycling, and improve effluent clarity [101]. Further, we detected frequent occurrences of flagellates belonging to *Kinetoplastida*, which are also recognized as efficient bacterivores. Together, these protistan groups likely exert selective predation pressure on bacterial communities through their diverse feeding strategies [102].

Together, these observations indicate that both viral infection and protistan predation act as important structuring forces within the prokaryotic community in the wastewater microbiome. The observed positive associations suggest that both viral and eukaryotic diversity may promote prokaryotic diversity. This supports the idea that the prokaryotic community is shaped not only by environmental factors but also by biotic interactions [24]. While our analysis does not allow for a direct identification of the underlying mechanisms, we cautiously interpret this pattern as being potentially driven by predation pressure in the case of microeukaryotes and host infection in the case of bacteriophages. These interactions could result in increased prokaryotic diversity and, in turn, influence viral and eukaryotic diversity as well.

A change in prokaryotic diversity also determines ARG diversity. In our data, we observed a significant negative correlation between prokaryotic and ARG diversity. Such negative correlations were recently investigated by Klümper et al. [3], however, in structured soil environments and not wastewater environments. Nevertheless, our results point in a similar direction, supporting their idea that increased microbial diversity can function as a natural barrier to ARG proliferation. Beyond the role of prokaryotic diversity, bacteriophages have also been recognized as potential contributors to the persistence and dissemination of ARGs [103–105]. In our dataset, we detected a broad range of ARGs, covering resistance to 21 different antibiotic classes. Among them, ARGs conferring resistance to bacitracin, beta-lactam antibiotics, and multiple drugs were the most abundant. Many of these classes have also been detected in other metagenomic studies of wastewater samples [7, 106]. We identified several significant correlations between viral taxa of the *Caudoviricetes* and ARG classes and additionally demonstrated a significant positive association between viral and ARG diversity, indicating that ARG diversity may vary in response to shifts in viral diversity. Additionally, we observed a significant positive association of eukaryotic diversity with ARG diversity. We suggest that this pattern may be driven by increased protistan predation, which was reported to shift bacterial communities towards higher ARG abundance and diversity in soil environments [4].

## Conclusion

This study provides evidence that the diversity of ARGs in the wastewater microbiome is closely linked to shifts in prokaryotic diversity, which is shaped by seasonal dynamics affecting viral and eukaryotic communities. With this, we underline the importance of including a broader view across microbial trophic levels to fully capture microbial dynamics in wastewater ecosystems, including those related to ARGs. By emphasizing the roles of viruses and microeukaryotes in shaping bacterial diversity, we contribute to a better understanding of the processes underlying ARG diversity and variation in wastewater environments. While our findings reveal significant associations across microbial trophic levels, we acknowledge that correlation-based analyses cannot unambiguously establish causality. Future experimental work using controlled microcosms with manipulated viral or protist communities will be essential to disentangle direct effects and validate the inferred ecological interactions.

## Supplementary Information

Supplementary Material 1.

## Data Availability

The code for the presented analyses is available over GitHub under the following link: (https://github.com/AWeiss17/The-Role-of-the-Viral-Community-Composition-in-Seasonal-Dynamics-of-the-Wastewater-Microbiome).

## Abbreviations

WWTPs: Wastewater treatment plants
ARGs: Antibiotic resistance genes
HGT: Horizontal gene transfer
SAR: Sequence Read Archive
OTU: Operational taxonomic unit
HMM: Hidden Markov model
SARG: Structured Antibiotic Resistance Gene
rRNA: Ribosomal RNA
NMDS: Non-metric multidimensional scaling
PCoA: Principal coordinate analysis
PERMANOVA: Permutational multivariate analysis of variance
SSU: Small subunit
SD: Standard deviation
