# Supplementary material for: Viral and eukaryotic drivers of prokaryotic and antibiotic resistance gene diversity in wastewater microbiomes
Source: Microbiome. 2026 Jan 10;14:24. doi: 10.1186/s40168-025-02307-3 (PMC12805738; doi:10.1186/s40168-025-02307-3)
Supplement: Supplementary file 2 — Supplementary Material 1. [file 40168_2025_2307_MOESM1_ESM.docx]

**Supplementary information**

“ Viral and Eukaryotic Drivers of Prokaryotic and Antibiotic Resistance Gene Diversity in Wastewater Microbiomes”

**Authors**

Antonia Weiss^a^, Alan Xavier Elena^b^, Uli Klümper^b^, and Kenneth Dumack^a,c^

**Corresponding author** Kenneth Dumack

Phone: +49-(0)261 287 - 2380

^a^ University of Cologne, Terrestrial Ecology, Institute of Zoology, Zülpicher Str. 47b, 50674 Köln, Germany

^b^ Technische Universität Dresden, Institute for Hydrobiology, 01217 Dresden, Germany

^c^ University of Koblenz, Aquatic Ecosystem Analyses, Institute for Mathematics / Natural Science, 56070 Koblenz, Germany

[aweiss12@smail.uni-koeln.de](mailto:aweiss12@smail.uni-koeln.de), [alan.elena@tu-dresden.de](mailto:alan.elena@tu-dresden.de), [uli.kluemper@tu-dresden.de](mailto:uli.kluemper@tu-dresden.de), [kenneth.dumack@uni-koblenz.de](mailto:kenneth.dumack@uni-koblenz.de)

1. *Rarefaction curves*


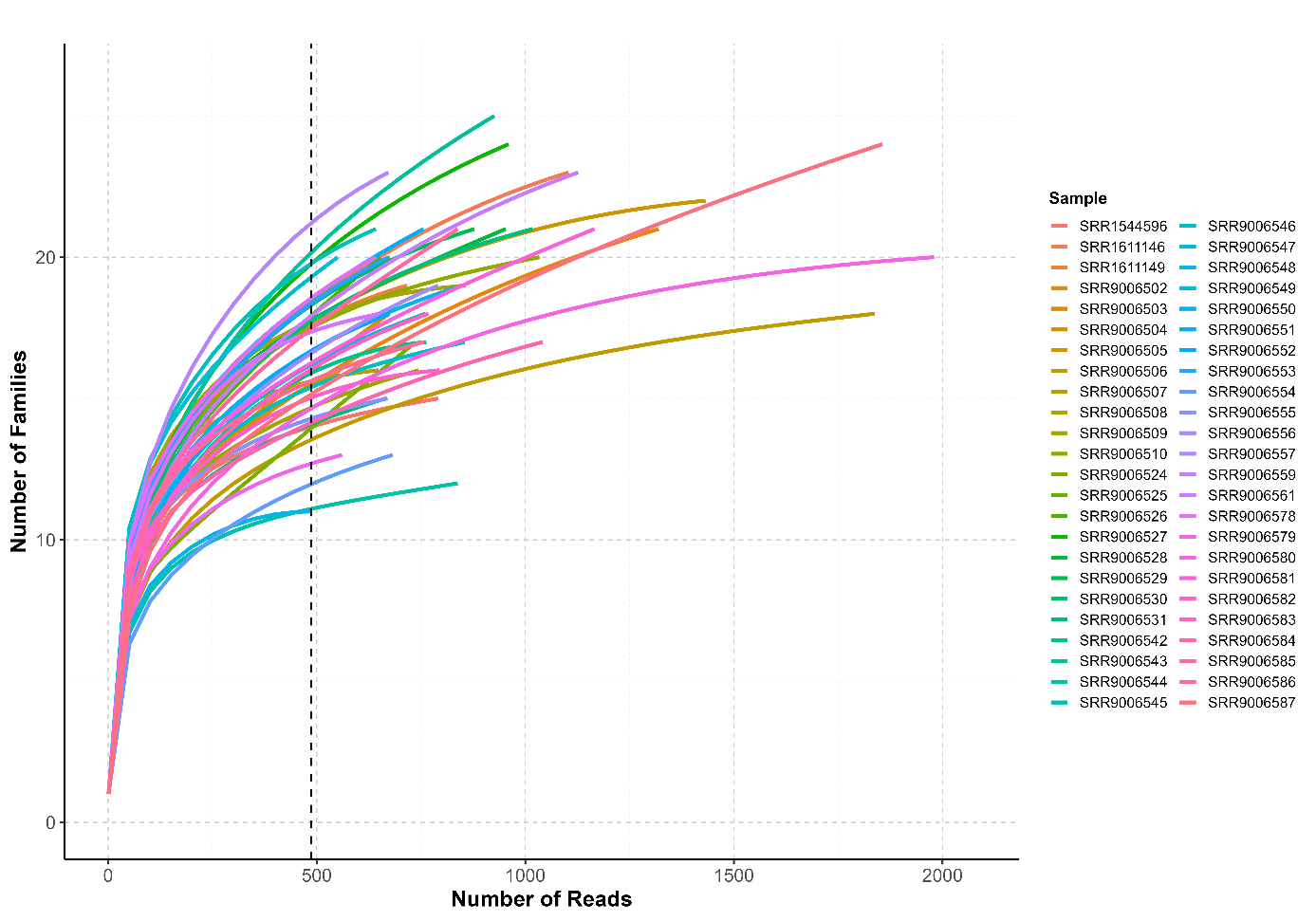


**Figure S1.** **Rarefaction curves of 48 metagenomic samples.** The number of detected viral families (y-axis) is plotted against the number of reads (x-axis). Three samples previously identified as outliers were excluded. The vertical dashed line indicates the rarefaction threshold at 486 reads, which represents the lowest sequencing depth across samples and was used for normalization of the dataset.


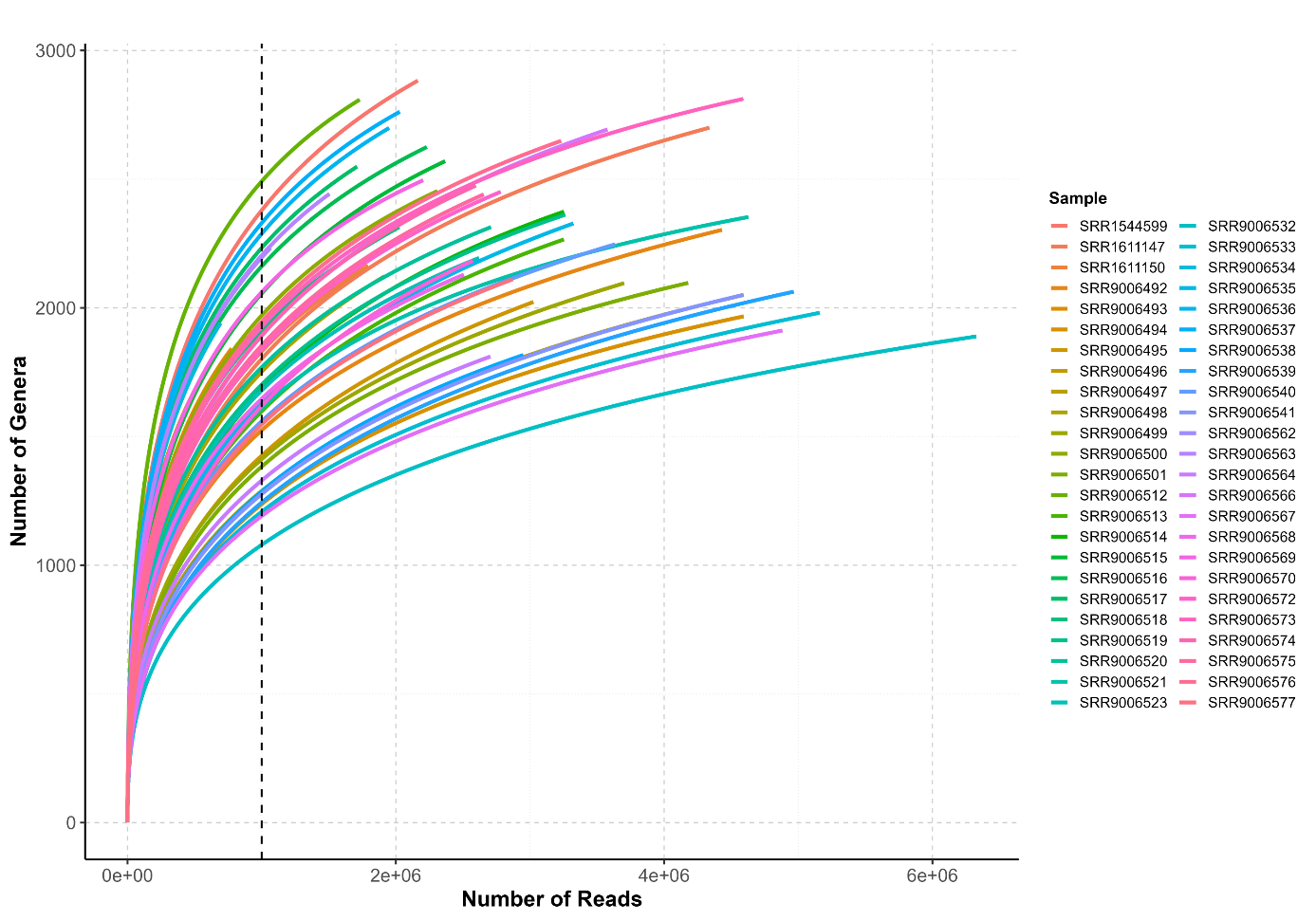


**Figure S2.** **Rarefaction curves of 48 metatranscriptomic samples.** The number of detected prokaryotic and eukaryotic genera (y-axis) is plotted against the number of reads (x-axis). Three samples previously identified as outliers were excluded. The vertical dashed line indicates the rarefaction threshold at 1,066,681 reads, which represents the lowest sequencing depth across samples and was used for normalization of the dataset.

2. *Time-decay analysis*


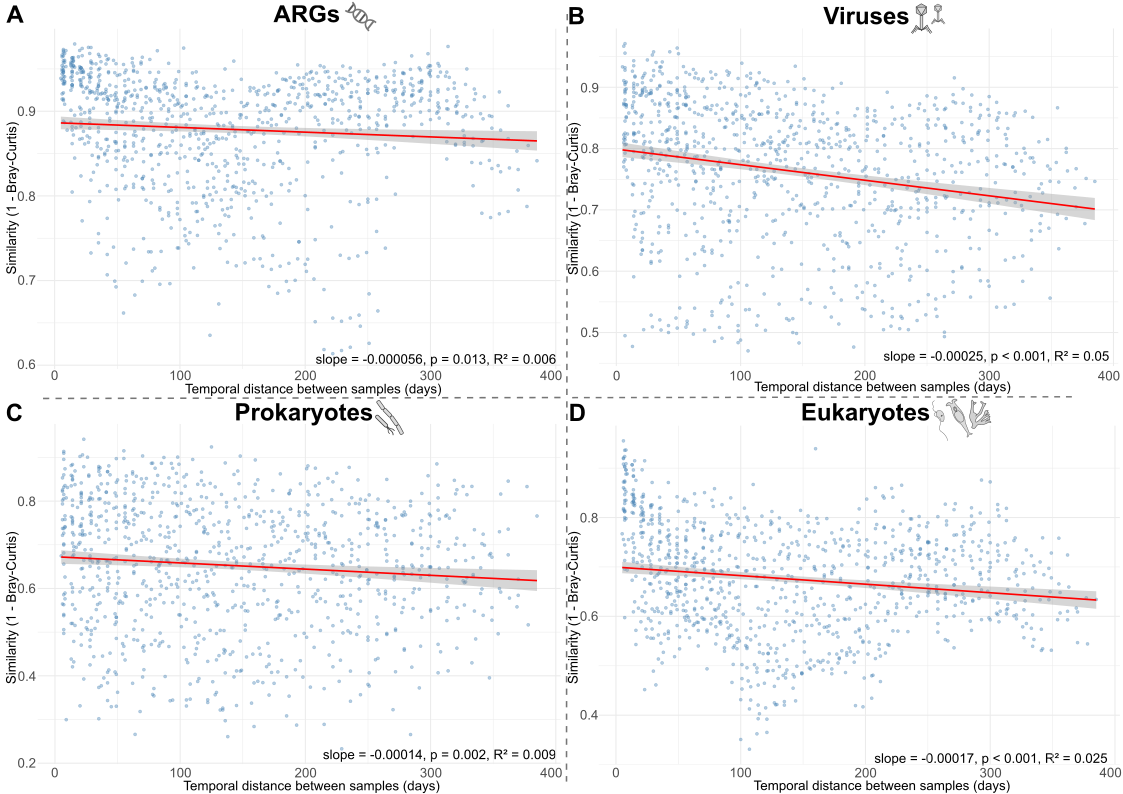


**Figure S3. Overview of Bray-Curtis dissimilarity time-decay analyses.** Time-decay relationships for (A) ARGs, (B) the viral, (C) the prokaryotic, and (D) the eukaryotic community. Bray-Curtis similarities (1 - Bray-Curtis dissimilarity) between all pairwise sample comparisons were plotted against the temporal distance between sampling dates (in days). Linear regressions (red lines) with 95% confidence intervals (gray shaded areas) were fitted to assess community turnover through time. The slope, *p*-value, and R²-value for each regression line are shown in the corner of the panel.

3. *PCoA*

**
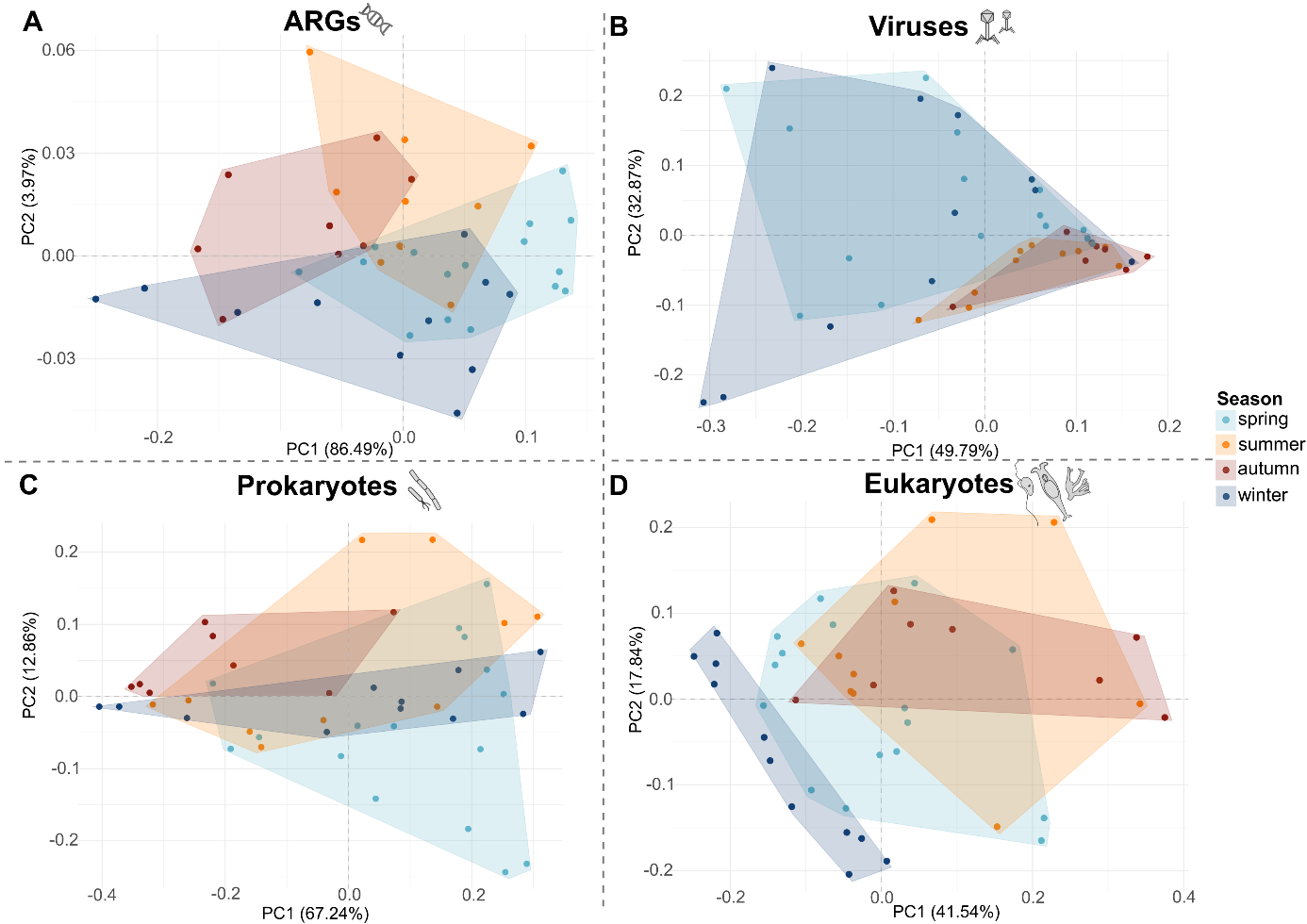
**

**Figure S4.** **Principal Coordinate Analysis (PCoA) plots based on Bray-Curtis dissimilarities across seasons (A-D).** Each dot represents one sample, color-coded by season spring (light blue), summer (yellow), autumn (red), and winter (dark blue). Polygons encompass samples from the same season. The plots display **(A)** ARG structure, **(B)** viral community structure, **(C)** prokaryotic community structure, and **(D)** eukaryotic community structure.

4. *PERMANOVA*

**
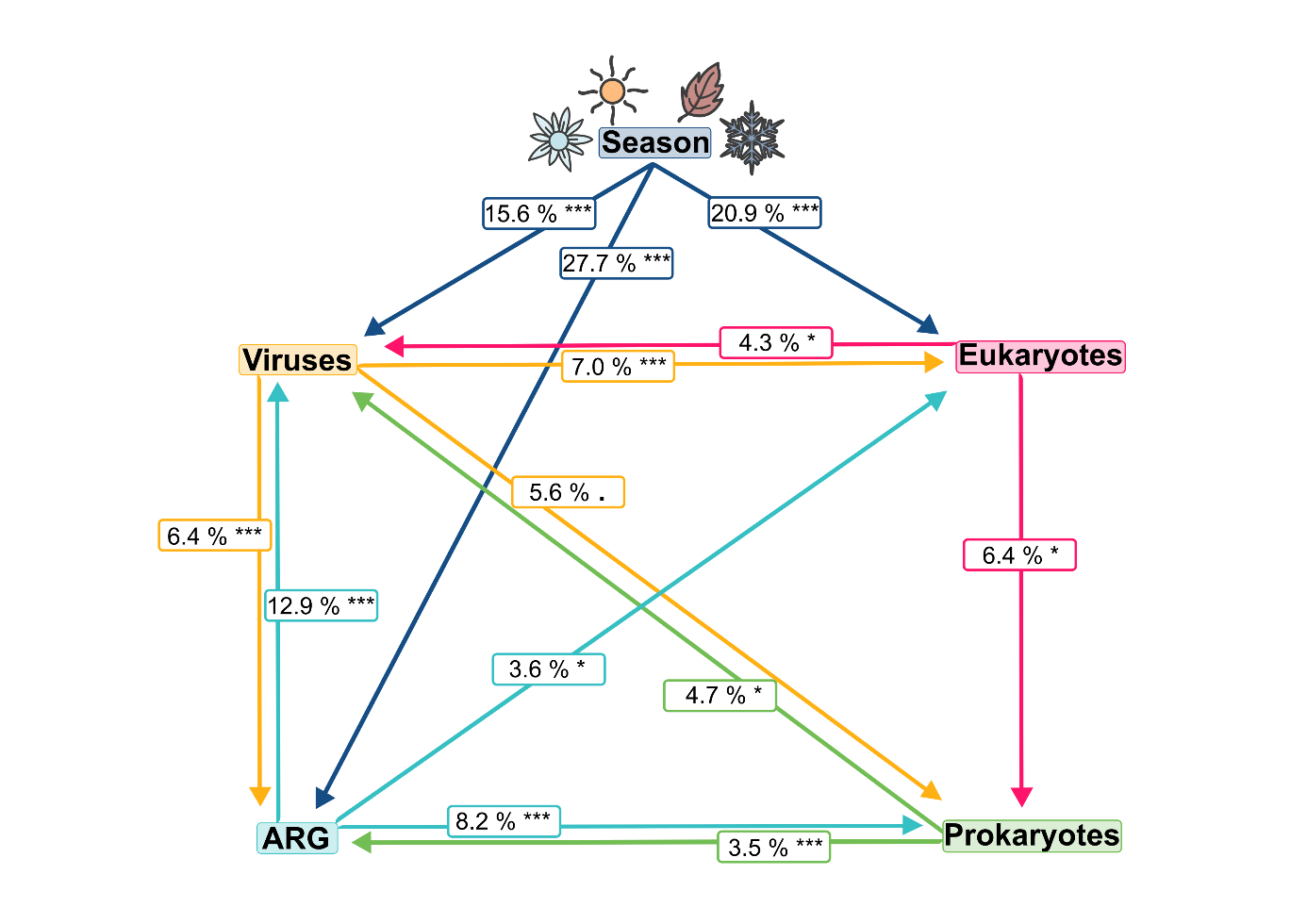
**

**Figure S5. Overview of significant influences based on PERMANOVA results.** Arrows indicate the direction of tested effects between season (dark blue), viruses (yellow), eukaryotes (pink), prokaryotes (green), and ARGs (light blue). For viral, prokaryotic, eukaryotic, and ARG communities, the structure (PCoA1) was used as a predictor in this analysis. Values represent the proportion of explained variance (R²). Significance levels are indicated as **p* ≤ 0.05, ***p* ≤ 0.01, ****p* ≤ 0.001. The effect of the viral community on the prokaryotic community is shown for completeness, although it did not reach statistical significance.

5. *Spearman correlation*


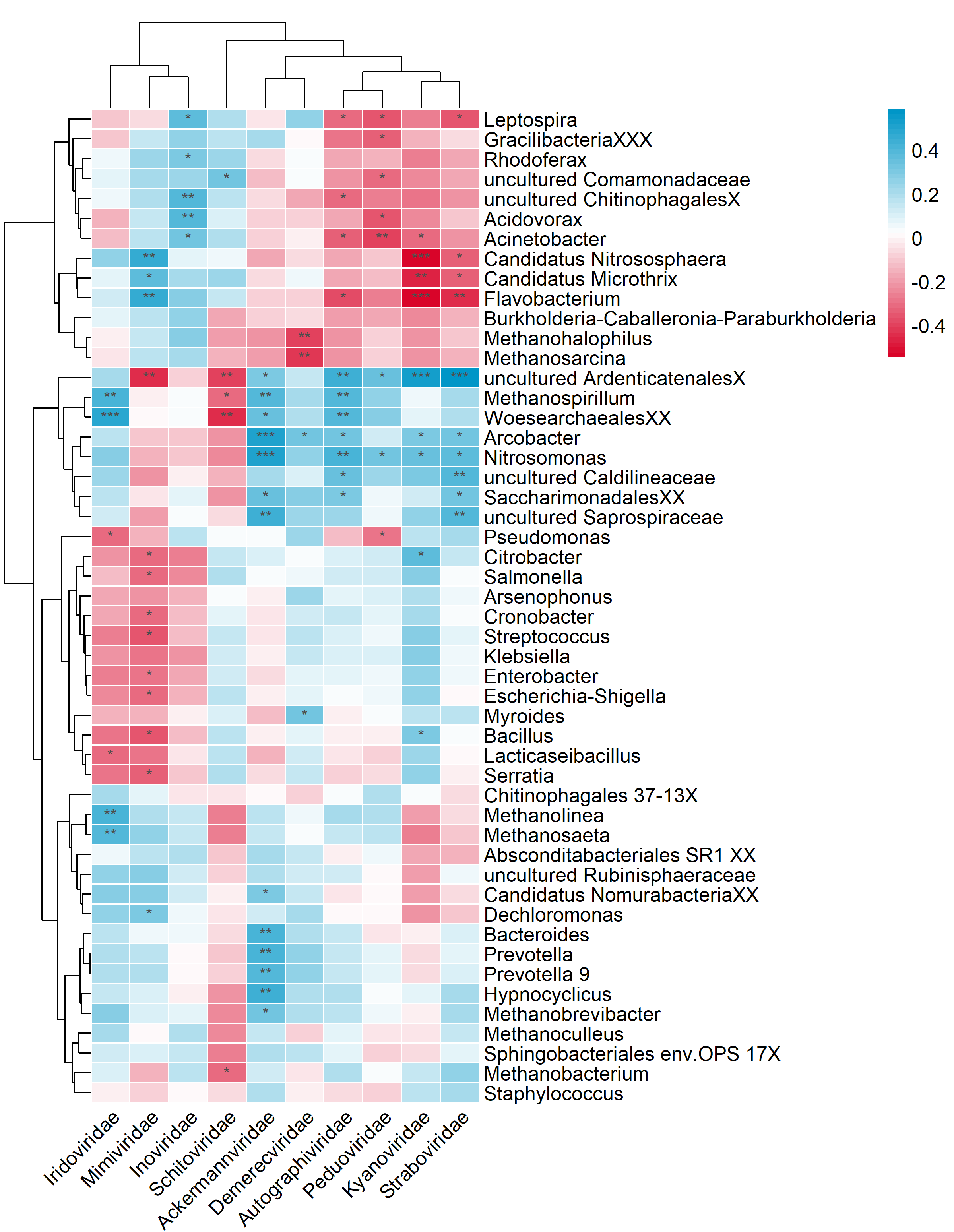


**Figure S6: Correlations and associations between viruses and prokaryotes.** Spearman correlation heatmaps showing associations between prokaryotic genera (y-axis) and viral families (x-axis). Correlation coefficients are color-coded (blue = positive, red = negative), and significance is indicated with asterisks **p* ≤ 0.05, ***p* ≤ 0.01, ****p* ≤ 0.001.

6. *Sample overview*

**Table S1. Overview of all 51 samples used in this study.** Displayed are the corresponding sampling dates (Date), internal sample identifiers (Sample ID), and the associated Sequence Read Archive accession numbers for both the metagenomic (SRR ID MG) and metatranscriptomic (SRR ID MT) datasets.

| **Date** | **Sample ID** | **SRR ID MG** | **SRR ID MT** |
| --- | --- | --- | --- |
| 21.03.2011 | D37 | SRR9006558 | SRR9006571 |
| 29.03.2011 | D02 | SRR9006511 | SRR9006522 |
| 05.04.2011 | D39 | SRR9006560 | SRR9006565 |
| 14.04.2011 | D24 | SRR9006548 | SRR9006519 |
| 21.04.2011 | D25 | SRR9006547 | SRR9006520 |
| 29.04.2011 | D35 | SRR9006557 | SRR9006568 |
| 06.05.2011 | D46 | SRR9006526 | SRR9006577 |
| 13.05.2011 | D13 | SRR9006582 | SRR9006495 |
| 20.05.2011 | D40 | SRR9006561 | SRR9006564 |
| 27.05.2011 | D33 | SRR9006555 | SRR9006513 |
| 03.06.2011 | D06 | SRR9006507 | SRR9006535 |
| 09.06.2011 | D27 | SRR9006545 | SRR9006514 |
| 17.06.2011 | D42 | SRR9006553 | SRR9006566 |
| 24.06.2011 | D03 | SRR9006508 | SRR9006536 |
| 01.07.2011 | D47 | SRR9006529 | SRR9006574 |
| 08.07.2011 | D18 | SRR9006579 | SRR9006498 |
| 05.08.2011 | D45 | SRR9006527 | SRR9006576 |
| 11.08.2011 | D51 | SRR9006530 | SRR9006573 |
| 19.08.2011 | D43 | SRR9006525 | SRR9006563 |
| 29.08.2011 | D34 | SRR9006556 | SRR9006569 |
| 05.10.2011 | D49 | SRR1611149 | SRR9006517 |
| 12.10.2011 | D32 | SRR1544596 | SRR9006537 |
| 05.09.2011 | D30 | SRR9006542 | SRR9006512 |
| 12.09.2011 | D04 | SRR9006509 | SRR9006516 |
| 19.09.2011 | D31 | SRR9006554 | SRR1611150 |
| 28.09.2011 | D29 | SRR9006543 | SRR1544599 |
| 02.11.2011 | D23 | SRR9006549 | SRR9006518 |
| 07.11.2011 | D44 | SRR9006524 | SRR9006562 |
| 16.11.2011 | D20 | SRR9006587 | SRR9006496 |
| 23.11.2011 | D15 | SRR9006580 | SRR9006493 |
| 29.11.2011 | D05 | SRR9006506 | SRR9006534 |
| 21.12.2011 | D28 | SRR9006544 | SRR9006515 |
| 28.12.2011 | D09 | SRR9006502 | SRR9006538 |
| 03.01.2012 | D38 | SRR9006559 | SRR9006570 |
| 11.01.2012 | D36 | SRR1611146 | SRR1611147 |
| 19.01.2012 | D12 | SRR9006585 | SRR9006533 |
| 25.01.2012 | D10 | SRR9006503 | SRR9006539 |
| 01.02.2012 | D14 | SRR9006583 | SRR9006494 |
| 08.02.2012 | D08 | SRR9006505 | SRR9006541 |
| 14.02.2012 | D26 | SRR9006546 | SRR9006521 |
| 23.02.2012 | D21 | SRR9006551 | SRR9006501 |
| 29.02.2012 | D19 | SRR9006586 | SRR9006497 |
| 08.03.2012 | D17 | SRR9006578 | SRR9006499 |
| 14.03.2012 | D41 | SRR9006552 | SRR9006567 |
| 22.03.2012 | D11 | SRR9006584 | SRR9006532 |
| 28.03.2012 | D16 | SRR9006581 | SRR9006492 |
| 04.04.2012 | D07 | SRR9006504 | SRR9006540 |
| 10.04.2012 | D22 | SRR9006550 | SRR9006500 |
| 17.04.2012 | D01 | SRR9006510 | SRR9006523 |
| 25.04.2012 | D50 | SRR9006531 | SRR9006572 |
| 03.05.2012 | D48 | SRR9006528 | SRR9006575 |

7. *Kraken2 output*

**Table S2.** **Overview of viral read classification results from Kraken2 for all 51 metagenomic samples.** Displayed are Sequence Read Archive identifiers (SRR ID), the number of reads classified as viral (Reads classified), the number of unclassified reads (Reads unclassified), and their corresponding proportions [%] based on total read counts per sample, using the RefSeq database from NCBI.

| **SRR ID** | **Reads classified** | **[%]** | **Reads unclassified** | **[%]** |
| --- | --- | --- | --- | --- |
| SRR9006558 | 63187 | 0.21 | 30459530 | 99.79 |
| SRR9006511 | 56155 | 0.22 | 25923171 | 99.78 |
| SRR9006560 | 60448 | 0.24 | 25258263 | 99.76 |
| SRR9006548 | 61522 | 0.24 | 25995645 | 99.76 |
| SRR9006547 | 39310 | 0.21 | 18454056 | 99.79 |
| SRR9006557 | 52924 | 0.21 | 25508333 | 99.79 |
| SRR9006526 | 51317 | 0.22 | 23418411 | 99.78 |
| SRR9006582 | 64742 | 0.23 | 27826140 | 99.77 |
| SRR9006561 | 49781 | 0.21 | 23115348 | 99.79 |
| SRR9006555 | 58064 | 0.22 | 26810444 | 99.78 |
| SRR9006507 | 53093 | 0.21 | 25335894 | 99.79 |
| SRR9006545 | 53849 | 0.22 | 24444993 | 99.78 |
| SRR9006553 | 55150 | 0.23 | 24126281 | 99.77 |
| SRR9006508 | 67181 | 0.24 | 27770060 | 99.76 |
| SRR9006529 | 63874 | 0.24 | 26105438 | 99.76 |
| SRR9006579 | 49593 | 0.23 | 21096744 | 99.77 |
| SRR9006527 | 64239 | 0.24 | 26507212 | 99.76 |
| SRR9006530 | 58420 | 0.23 | 25249080 | 99.77 |
| SRR9006525 | 51505 | 0.22 | 22876863 | 99.78 |
| SRR9006556 | 52989 | 0.20 | 25919772 | 99.80 |
| SRR9006542 | 58936 | 0.22 | 27237555 | 99.78 |
| SRR9006509 | 58635 | 0.24 | 24405363 | 99.76 |
| SRR9006554 | 54044 | 0.24 | 22470802 | 99.76 |
| SRR9006543 | 60377 | 0.24 | 25456866 | 99.76 |
| SRR9006549 | 37696 | 0.22 | 16945706 | 99.78 |
| SRR9006524 | 56214 | 0.24 | 23340817 | 99.76 |
| SRR9006587 | 76197 | 0.26 | 28913298 | 99.74 |
| SRR9006580 | 63819 | 0.28 | 23139926 | 99.72 |
| SRR9006506 | 59853 | 0.27 | 22083136 | 99.73 |
| SRR9006544 | 69157 | 0.26 | 26173560 | 99.74 |
| SRR9006502 | 58070 | 0.24 | 24073263 | 99.76 |
| SRR9006559 | 53151 | 0.24 | 21772816 | 99.76 |
| SRR9006585 | 58071 | 0.25 | 23567132 | 99.75 |
| SRR9006503 | 56433 | 0.21 | 26523696 | 99.79 |
| SRR9006583 | 46123 | 0.22 | 20524670 | 99.78 |
| SRR9006505 | 70040 | 0.25 | 27768867 | 99.75 |
| SRR9006546 | 61551 | 0.24 | 25567854 | 99.76 |
| SRR9006551 | 49673 | 0.23 | 21843065 | 99.77 |
| SRR9006586 | 58498 | 0.22 | 26388392 | 99.78 |
| SRR9006578 | 47304 | 0.22 | 21306588 | 99.78 |
| SRR9006552 | 59368 | 0.22 | 26934966 | 99.78 |
| SRR9006584 | 64128 | 0.24 | 26397625 | 99.76 |
| SRR9006581 | 58671 | 0.23 | 25192062 | 99.77 |
| SRR9006504 | 30851 | 0.22 | 14303068 | 99.78 |
| SRR9006550 | 59846 | 0.21 | 28346091 | 99.79 |
| SRR9006510 | 63552 | 0.24 | 26206885 | 99.76 |
| SRR9006531 | 52548 | 0.25 | 21145998 | 99.75 |
| SRR9006528 | 58789 | 0.24 | 24075492 | 99.76 |
| SRR1611149 | 55396 | 0.22 | 25167966 | 99.78 |
| SRR1544596 | 61457 | 0.24 | 25521322 | 99.76 |
| SRR1611146 | 65171 | 0.22 | 29699454 | 99.78 |

8. *Raw sequencing output*

**Table S3.** **Overview of raw sequencing output for the selected samples used in this study.** Displayed are the Sequence Read Archive identifiers (SRR ID), the total number of raw reads generated per sample (Reads), the library type (Library Source: metagenomic or metatranscriptomic), and the corresponding sampling date (Date). Raw read counts represent the unprocessed output directly after sequencing.

| **SRR ID** | **Reads** | **Library Source** | **Date** |
| --- | --- | --- | --- |
| SRR9006558 | 35,999,747 | METAGENOMIC | 21.03.2011 |
| SRR9006571 | 35,975,301 | METATRANSCRIPTOMIC | 21.03.2011 |
| SRR9006511 | 34,135,175 | METAGENOMIC | 29.03.2011 |
| SRR9006522 | 33,912,215 | METATRANSCRIPTOMIC | 29.03.2011 |
| SRR9006560 | 28,908,243 | METAGENOMIC | 05.04.2011 |
| SRR9006565 | 38,592,454 | METATRANSCRIPTOMIC | 05.04.2011 |
| SRR9006519 | 36,395,674 | METATRANSCRIPTOMIC | 14.04.2011 |
| SRR9006548 | 29,187,268 | METAGENOMIC | 14.04.2011 |
| SRR9006520 | 33,954,127 | METATRANSCRIPTOMIC | 21.04.2011 |
| SRR9006547 | 25,320,222 | METAGENOMIC | 21.04.2011 |
| SRR9006557 | 30,371,838 | METAGENOMIC | 29.04.2011 |
| SRR9006568 | 29,681,622 | METATRANSCRIPTOMIC | 29.04.2011 |
| SRR9006526 | 28,122,705 | METAGENOMIC | 06.05.2011 |
| SRR9006577 | 34,116,436 | METATRANSCRIPTOMIC | 06.05.2011 |
| SRR9006495 | 35,894,482 | METATRANSCRIPTOMIC | 13.05.2011 |
| SRR9006582 | 31,173,943 | METAGENOMIC | 13.05.2011 |
| SRR9006561 | 25,651,909 | METAGENOMIC | 20.05.2011 |
| SRR9006564 | 27,707,752 | METATRANSCRIPTOMIC | 20.05.2011 |
| SRR9006513 | 27,369,514 | METATRANSCRIPTOMIC | 27.05.2011 |
| SRR9006555 | 30,235,782 | METAGENOMIC | 27.05.2011 |
| SRR9006507 | 30,216,637 | METAGENOMIC | 03.06.2011 |
| SRR9006535 | 32,430,876 | METATRANSCRIPTOMIC | 03.06.2011 |
| SRR9006514 | 30,331,588 | METATRANSCRIPTOMIC | 09.06.2011 |
| SRR9006545 | 27,497,422 | METAGENOMIC | 09.06.2011 |
| SRR9006553 | 27,303,250 | METAGENOMIC | 17.06.2011 |
| SRR9006566 | 34,544,359 | METATRANSCRIPTOMIC | 17.06.2011 |
| SRR9006508 | 32,347,395 | METAGENOMIC | 24.06.2011 |
| SRR9006536 | 32,699,565 | METATRANSCRIPTOMIC | 24.06.2011 |
| SRR9006529 | 30,109,054 | METAGENOMIC | 01.07.2011 |
| SRR9006574 | 36,766,788 | METATRANSCRIPTOMIC | 01.07.2011 |
| SRR9006498 | 31,924,022 | METATRANSCRIPTOMIC | 08.07.2011 |
| SRR9006579 | 23,391,679 | METAGENOMIC | 08.07.2011 |
| SRR9006527 | 29,930,665 | METAGENOMIC | 05.08.2011 |
| SRR9006576 | 35,578,306 | METATRANSCRIPTOMIC | 05.08.2011 |
| SRR9006530 | 29,852,842 | METAGENOMIC | 11.08.2011 |
| SRR9006573 | 44,174,409 | METATRANSCRIPTOMIC | 11.08.2011 |
| SRR9006525 | 26,075,473 | METAGENOMIC | 19.08.2011 |
| SRR9006563 | 27,171,859 | METATRANSCRIPTOMIC | 19.08.2011 |
| SRR9006556 | 30,403,109 | METAGENOMIC | 29.08.2011 |
| SRR9006569 | 28,421,236 | METATRANSCRIPTOMIC | 29.08.2011 |
| SRR9006517 | 29,515,165 | METATRANSCRIPTOMIC | 05.09.2011 |
| SRR9006542 | 30,532,662 | METAGENOMIC | 05.09.2011 |
| SRR9006509 | 29,014,224 | METAGENOMIC | 12.09.2011 |
| SRR9006537 | 35,007,981 | METATRANSCRIPTOMIC | 12.09.2011 |
| SRR9006512 | 30,499,924 | METATRANSCRIPTOMIC | 19.09.2011 |
| SRR9006554 | 25,456,037 | METAGENOMIC | 19.09.2011 |
| SRR9006516 | 33,708,983 | METATRANSCRIPTOMIC | 28.09.2011 |
| SRR9006543 | 29,839,175 | METAGENOMIC | 28.09.2011 |
| SRR1611149 | 29,042,305 | METAGENOMIC | 05.10.2011 |
| SRR1611150 | 29,639,609 | METATRANSCRIPTOMIC | 05.10.2011 |
| SRR1544596 | 28,460,125 | METAGENOMIC | 12.10.2011 |
| SRR1544599 | 31,894,222 | METATRANSCRIPTOMIC | 12.10.2011 |
| SRR9006518 | 32,127,592 | METATRANSCRIPTOMIC | 02.11.2011 |
| SRR9006549 | 23,448,618 | METAGENOMIC | 02.11.2011 |
| SRR9006524 | 27,963,482 | METAGENOMIC | 07.11.2011 |
| SRR9006562 | 23,803,138 | METATRANSCRIPTOMIC | 07.11.2011 |
| SRR9006496 | 33,469,570 | METATRANSCRIPTOMIC | 16.11.2011 |
| SRR9006587 | 32,139,021 | METAGENOMIC | 16.11.2011 |
| SRR9006493 | 35,418,302 | METATRANSCRIPTOMIC | 23.11.2011 |
| SRR9006580 | 26,973,299 | METAGENOMIC | 23.11.2011 |
| SRR9006506 | 25,621,580 | METAGENOMIC | 29.11.2011 |
| SRR9006534 | 32,499,996 | METATRANSCRIPTOMIC | 29.11.2011 |
| SRR9006515 | 34,336,596 | METATRANSCRIPTOMIC | 21.12.2011 |
| SRR9006544 | 29,329,592 | METAGENOMIC | 21.12.2011 |
| SRR9006502 | 31,138,147 | METAGENOMIC | 28.12.2011 |
| SRR9006538 | 33,883,001 | METATRANSCRIPTOMIC | 28.12.2011 |
| SRR9006559 | 24,493,806 | METAGENOMIC | 03.01.2012 |
| SRR9006570 | 23,571,029 | METATRANSCRIPTOMIC | 03.01.2012 |
| SRR1611146 | 34,928,806 | METAGENOMIC | 11.01.2012 |
| SRR1611147 | 37,853,692 | METATRANSCRIPTOMIC | 11.01.2012 |
| SRR9006533 | 32,078,032 | METATRANSCRIPTOMIC | 19.01.2012 |
| SRR9006585 | 26,256,782 | METAGENOMIC | 19.01.2012 |
| SRR9006503 | 32,576,299 | METAGENOMIC | 25.01.2012 |
| SRR9006539 | 35,356,100 | METATRANSCRIPTOMIC | 25.01.2012 |
| SRR9006494 | 33,583,347 | METATRANSCRIPTOMIC | 01.02.2012 |
| SRR9006583 | 26,995,145 | METAGENOMIC | 01.02.2012 |
| SRR9006505 | 33,462,713 | METAGENOMIC | 08.02.2012 |
| SRR9006541 | 32,414,295 | METATRANSCRIPTOMIC | 08.02.2012 |
| SRR9006521 | 31,735,326 | METATRANSCRIPTOMIC | 14.02.2012 |
| SRR9006546 | 29,242,844 | METAGENOMIC | 14.02.2012 |
| SRR9006501 | 34,301,246 | METATRANSCRIPTOMIC | 23.02.2012 |
| SRR9006551 | 24,350,474 | METAGENOMIC | 23.02.2012 |
| SRR9006497 | 36,694,504 | METATRANSCRIPTOMIC | 29.02.2012 |
| SRR9006586 | 32,574,065 | METAGENOMIC | 29.02.2012 |
| SRR9006499 | 30,362,735 | METATRANSCRIPTOMIC | 08.03.2012 |
| SRR9006578 | 23,876,138 | METAGENOMIC | 08.03.2012 |
| SRR9006552 | 30,626,165 | METAGENOMIC | 14.03.2012 |
| SRR9006567 | 27,396,429 | METATRANSCRIPTOMIC | 14.03.2012 |
| SRR9006532 | 35,677,196 | METATRANSCRIPTOMIC | 22.03.2012 |
| SRR9006584 | 29,495,784 | METAGENOMIC | 22.03.2012 |
| SRR9006492 | 36,820,589 | METATRANSCRIPTOMIC | 28.03.2012 |
| SRR9006581 | 28,186,198 | METAGENOMIC | 28.03.2012 |
| SRR9006504 | 21,699,617 | METAGENOMIC | 04.04.2012 |
| SRR9006540 | 34,514,784 | METATRANSCRIPTOMIC | 04.04.2012 |
| SRR9006500 | 34,097,677 | METATRANSCRIPTOMIC | 10.04.2012 |
| SRR9006550 | 33,671,265 | METAGENOMIC | 10.04.2012 |
| SRR9006510 | 32,692,858 | METAGENOMIC | 17.04.2012 |
| SRR9006523 | 33,418,152 | METATRANSCRIPTOMIC | 17.04.2012 |
| SRR9006531 | 24,359,112 | METAGENOMIC | 25.04.2012 |
| SRR9006572 | 32,882,193 | METATRANSCRIPTOMIC | 25.04.2012 |
| SRR9006528 | 27,267,336 | METAGENOMIC | 03.05.2012 |
| SRR9006575 | 32,450,531 | METATRANSCRIPTOMIC | 03.05.2012 |

9. *Environmental parameters*

**Table S4. Overview of environmental parameters measured for all 51 samples used in this study.** Displayed are the sampling date (Date), water temperature (°C), pH, season, and internal sample identifiers (Sample ID) for each sample from the metagenomic and metatranscriptomic datasets.

| **Date** | **Water temp.**  **[°C]** | **pH** | **Season** | **Sample ID** |
| --- | --- | --- | --- | --- |
| 21.03.2011 | 13.6 | 6.939166667 | spring | D37 |
| 29.03.2011 | 13.3 | 6.993333333 | spring | D02 |
| 05.04.2011 | 13.4 | 6.8725 | spring | D39 |
| 14.04.2011 | 14.5 | 6.98 | spring | D24 |
| 21.04.2011 | 15.7 | 6.975833333 | spring | D25 |
| 29.04.2011 | 15.7 | 6.95 | spring | D35 |
| 06.05.2011 | 16.1 | 6.976666667 | spring | D46 |
| 13.05.2011 | 17.4 | 6.934166667 | spring | D13 |
| 20.05.2011 | 17.6 | 6.951666667 | spring | D40 |
| 27.05.2011 | 17.5 | 7.015833333 | spring | D33 |
| 03.06.2011 | 18.3 | 6.684166667 | summer | D06 |
| 09.06.2011 | 18.3 | 6.6525 | summer | D27 |
| 17.06.2011 | 18.8 | 6.706666667 | summer | D42 |
| 24.06.2011 | 17.9 | 6.683333333 | summer | D03 |
| 01.07.2011 | 19.0 | 6.649166667 | summer | D47 |
| 08.07.2011 | 19.8 | 6.785 | summer | D18 |
| 05.08.2011 | 20.0 | 6.6 | summer | D45 |
| 11.08.2011 | 18.9 | 6.640833333 | summer | D51 |
| 19.08.2011 | 20.1 | 6.771666667 | summer | D43 |
| 29.08.2011 | 19.0 | 6.650833333 | summer | D34 |
| 05.09.2011 | 20.2 | 6.723333333 | autumn | D30 |
| 12.09.2011 | 19.2 | 6.648333333 | autumn | D04 |
| 19.09.2011 | 18.4 | 6.618333333 | autumn | D31 |
| 28.09.2011 | 19.5 | 6.7025 | autumn | D29 |
| 05.10.2011 | 19.7 | 6.7175 | autumn | D49 |
| 12.10.2011 | 17.9 | 6.66 | autumn | D32 |
| 02.11.2011 | 17.2 | 6.725833333 | autumn | D23 |
| 07.11.2011 | 17.2 | 6.660833333 | autumn | D44 |
| 16.11.2011 | 15.9 | 6.714166667 | autumn | D20 |
| 23.11.2011 | 16.2 | 6.716666667 | autumn | D15 |
| 29.11.2011 | 15.7 | 6.730833333 | autumn | D05 |
| 21.12.2011 | 11.1 | 6.801666667 | winter | D28 |
| 28.12.2011 | 12.3 | 6.806666667 | winter | D09 |
| 03.01.2012 | 11.6 | 6.873333333 | winter | D38 |
| 11.01.2012 | 11.5 | 6.863333333 | winter | D36 |
| 19.01.2012 | 12.1 | 6.813333333 | winter | D12 |
| 25.01.2012 | 10.7 | 6.859166667 | winter | D10 |
| 01.02.2012 | 10.7 | 6.813333333 | winter | D14 |
| 08.02.2012 | 10.6 | 6.704166667 | winter | D08 |
| 14.02.2012 | 11.2 | 6.675833333 | winter | D26 |
| 23.02.2012 | 10.6 | 6.734166667 | winter | D21 |
| 29.02.2012 | 11.8 | 6.830833333 | winter | D19 |
| 08.03.2012 | 11.0 | 6.748333333 | spring | D17 |
| 14.03.2012 | 12.2 | 6.986666667 | spring | D41 |
| 22.03.2012 | 12.8 | 6.9575 | spring | D11 |
| 28.03.2012 | 14.5 | 6.9925 | spring | D16 |
| 04.04.2012 | 14.0 | 7.1675 | spring | D07 |
| 10.04.2012 | 13.2 | 7.256666667 | spring | D22 |
| 17.04.2012 | 13.9 | 6.985 | spring | D01 |
| 25.04.2012 | 12.7 | 7.021666667 | spring | D50 |
| 03.05.2012 | 13.4 | 7.150833333 | spring | D48 |

10. *Spearman correlation coefficients*

**Table S5.** **Significant Spearman correlations between viral families and ARG classes.** Displayed are viral families and ARG classes with significant correlations, including Spearman correlation coefficient (cor.coeff) and corresponding *p*-value. Only statistically significant associations (*p* ≤ 0.05) are shown.

| **Viruses** | **ARGs** | **cor.coeff** | ***p*-value** |
| --- | --- | --- | --- |
| Iridoviridae | Aminoglycoside | -0.49504 | 0.000546 |
| Mimiviridae | Aminoglycoside | -0.30956 | 0.038516 |
| Autographiviridae | Aminoglycoside | -0.49455 | 0.000554 |
| Demerecviridae | Aminoglycoside | -0.41124 | 0.005009 |
| Schitoviridae | Aminoglycoside | 0.349454 | 0.018625 |
| Iridoviridae | Bacitracin | 0.618656 | 5.92E-06 |
| Mimiviridae | Bacitracin | 0.426452 | 0.003488 |
| Autographiviridae | Bacitracin | 0.430317 | 0.003173 |
| Peduoviridae | Bacitracin | 0.331543 | 0.026093 |
| Schitoviridae | Bacitracin | -0.33294 | 0.025432 |
| Inoviridae | Bacitracin | -0.30953 | 0.038537 |
| Iridoviridae | Beta_lactam | -0.65695 | 9.52E-07 |
| Mimiviridae | Beta_lactam | -0.43529 | 0.002804 |
| Autographiviridae | Beta_lactam | -0.5032 | 0.000426 |
| Peduoviridae | Beta_lactam | -0.447 | 0.002082 |
| Schitoviridae | Beta_lactam | 0.334667 | 0.024636 |
| Inoviridae | Beta_lactam | 0.346505 | 0.019713 |
| Iridoviridae | Macrolide-lincosamide-streptogramin | 0.626262 | 4.2E-06 |
| Mimiviridae | Macrolide-lincosamide-streptogramin | 0.4969 | 0.000516 |
| Autographiviridae | Macrolide-lincosamide-streptogramin | 0.3334 | 0.025218 |
| Iridoviridae | Multidrug | 0.424276 | 0.003677 |
| Ackermannviridae | Multidrug | 0.305945 | 0.040965 |
| Autographiviridae | Multidrug | 0.449793 | 0.001936 |
| Kyanoviridae | Multidrug | 0.385885 | 0.008844 |
| Peduoviridae | Multidrug | 0.347952 | 0.019172 |
| Schitoviridae | Multidrug | -0.36411 | 0.013939 |
| Straboviridae | Multidrug | 0.356781 | 0.016138 |
| Iridoviridae | Mupirocin | 0.483006 | 0.000778 |
| Mimiviridae | Mupirocin | 0.629618 | 3.6E-06 |
| Kyanoviridae | Mupirocin | -0.47827 | 0.000891 |
| Iridoviridae | Polymyxin | 0.576393 | 3.42E-05 |
| Mimiviridae | Polymyxin | 0.627969 | 3.88E-06 |
| Kyanoviridae | Polymyxin | -0.33633 | 0.023887 |
| Peduoviridae | Polymyxin | 0.312564 | 0.036579 |
| Iridoviridae | Rifamycin | 0.49921 | 0.000481 |
| Autographiviridae | Rifamycin | 0.503467 | 0.000423 |
| Schitoviridae | Rifamycin | -0.39713 | 0.00691 |
| Straboviridae | Rifamycin | 0.309845 | 0.038331 |
| Iridoviridae | Sulfonamide | -0.58803 | 2.16E-05 |
| Mimiviridae | Sulfonamide | -0.5064 | 0.000386 |
| Autographiviridae | Sulfonamide | -0.35208 | 0.017697 |
| Inoviridae | Sulfonamide | 0.308699 | 0.03909 |
| Iridoviridae | Tetracycline | 0.456948 | 0.001603 |
| Mimiviridae | Tetracycline | 0.528628 | 0.000189 |
| Autographiviridae | Tetracycline | 0.466364 | 0.001242 |

**Table S6.** **Significant Spearman correlations between viral families and prokaryotic orders.** Displayed are viral families and prokaryotic orders with significant correlations, including Spearman correlation coefficient (cor.coeff) and corresponding *p*-value. Only statistically significant associations (*p* ≤ 0.05) are shown.

| **Viruses** | **Prokaryotes** | **cor.coeff** | ***p*-value** |
| --- | --- | --- | --- |
| Iridoviridae | Bacillales | -0.2957 | 0.04859 |
| Mimiviridae | Bacillales | -0.35059 | 0.018218 |
| Kyanoviridae | Bacillales | 0.302922 | 0.043106 |
| Ackermannviridae | Bacteroidales | 0.421616 | 0.00392 |
| Ackermannviridae | Campylobacterales | 0.42446 | 0.00366 |
| Ackermannviridae | Chitinophagales | 0.380678 | 0.009887 |
| Mimiviridae | Enterobacterales | -0.30145 | 0.04418 |
| Mimiviridae | Flavobacteriales | 0.371109 | 0.012081 |
| Autographiviridae | Flavobacteriales | -0.40173 | 0.006232 |
| Kyanoviridae | Flavobacteriales | -0.4676 | 0.001201 |
| Straboviridae | Flavobacteriales | -0.29424 | 0.049765 |
| Inoviridae | Flavobacteriales | 0.377361 | 0.010606 |
| Iridoviridae | Lactobacillales | -0.29438 | 0.049654 |
| Mimiviridae | Lactobacillales | -0.34149 | 0.021686 |
| Peduoviridae | Leptospirales | -0.34887 | 0.018834 |
| Straboviridae | Leptospirales | -0.36035 | 0.015032 |
| Inoviridae | Leptospirales | 0.372218 | 0.011807 |
| Iridoviridae | Methanobacteriales | 0.308733 | 0.039068 |
| Ackermannviridae | Methanobacteriales | 0.360573 | 0.014966 |
| Autographiviridae | Methanobacteriales | 0.294382 | 0.049652 |
| Schitoviridae | Methanobacteriales | -0.34017 | 0.022232 |
| Straboviridae | Methanobacteriales | 0.354402 | 0.016913 |
| Iridoviridae | Methanomicrobiales | 0.393125 | 0.007552 |
| Ackermannviridae | Methanomicrobiales | 0.317518 | 0.033555 |
| Demerecviridae | Methanosarcinales | -0.38477 | 0.00906 |
| Kyanoviridae | Microtrichales | -0.4332 | 0.002954 |
| Mimiviridae | Nitrososphaerales | 0.459697 | 0.001489 |
| Kyanoviridae | Nitrososphaerales | -0.52717 | 0.000199 |
| Straboviridae | Nitrososphaerales | -0.32393 | 0.029953 |
| Autographiviridae | Pseudomonadales | -0.31914 | 0.032611 |
| Peduoviridae | Pseudomonadales | -0.41833 | 0.00424 |
| Inoviridae | Pseudomonadales | 0.329548 | 0.027062 |

**Table S7.** **Significant Spearman correlations between viral families and prokaryotic genera.** Displayed are viral families and prokaryotic genera with significant correlations, including Spearman correlation coefficient (cor.coeff) and corresponding *p*-value. Only statistically significant associations (*p* ≤ 0.05) are shown.

| **Viruses** | **Prokaryotes** | **cor.coeff** | ***p*-value** |
| --- | --- | --- | --- |
| Peduoviridae | Acidovorax | -0.34288 | 0.021124 |
| Inoviridae | Acidovorax | 0.398626 | 0.006683 |
| Autographiviridae | Acinetobacter | -0.33056 | 0.026566 |
| Kyanoviridae | Acinetobacter | -0.29811 | 0.046701 |
| Peduoviridae | Acinetobacter | -0.4039 | 0.005932 |
| Inoviridae | Acinetobacter | 0.324405 | 0.029697 |
| Ackermannviridae | Arcobacter | 0.501112 | 0.000454 |
| Autographiviridae | Arcobacter | 0.330495 | 0.026598 |
| Demerecviridae | Arcobacter | 0.332808 | 0.025495 |
| Kyanoviridae | Arcobacter | 0.307403 | 0.039963 |
| Straboviridae | Arcobacter | 0.328488 | 0.027589 |
| Mimiviridae | Bacillus | -0.35139 | 0.01794 |
| Kyanoviridae | Bacillus | 0.300221 | 0.045096 |
| Ackermannviridae | Bacteroides | 0.413482 | 0.004754 |
| Mimiviridae | Candidatus Microthrix | 0.365502 | 0.013551 |
| Kyanoviridae | Candidatus Microthrix | -0.46377 | 0.001333 |
| Straboviridae | Candidatus Microthrix | -0.32538 | 0.029181 |
| Mimiviridae | Candidatus Nitrososphaera | 0.459697 | 0.001489 |
| Kyanoviridae | Candidatus Nitrososphaera | -0.52717 | 0.000199 |
| Straboviridae | Candidatus Nitrososphaera | -0.32393 | 0.029953 |
| Ackermannviridae | Candidatus NomurabacteriaXX | 0.296818 | 0.047708 |
| Mimiviridae | Citrobacter | -0.31728 | 0.033694 |
| Kyanoviridae | Citrobacter | 0.366578 | 0.013258 |
| Mimiviridae | Cronobacter | -0.29822 | 0.046618 |
| Mimiviridae | Dechloromonas | 0.299473 | 0.04566 |
| Mimiviridae | Enterobacter | -0.29486 | 0.04927 |
| Mimiviridae | Escherichia-Shigella | -0.30429 | 0.042128 |
| Mimiviridae | Flavobacterium | 0.461412 | 0.001422 |
| Autographiviridae | Flavobacterium | -0.36582 | 0.013465 |
| Kyanoviridae | Flavobacterium | -0.54628 | 0.000104 |
| Straboviridae | Flavobacterium | -0.43671 | 0.002706 |
| Peduoviridae | GracilibacteriaXXX | -0.32831 | 0.027676 |
| Ackermannviridae | Hypnocyclicus | 0.44106 | 0.002424 |
| Iridoviridae | Lacticaseibacillus | -0.31138 | 0.037335 |
| Autographiviridae | Leptospira | -0.30435 | 0.042083 |
| Peduoviridae | Leptospira | -0.36028 | 0.015055 |
| Straboviridae | Leptospira | -0.35929 | 0.015353 |
| Inoviridae | Leptospira | 0.375832 | 0.010951 |
| Schitoviridae | Methanobacterium | -0.30164 | 0.044038 |
| Ackermannviridae | Methanobrevibacter | 0.33273 | 0.025531 |
| Demerecviridae | Methanohalophilus | -0.40959 | 0.005206 |
| Iridoviridae | Methanolinea | 0.417331 | 0.004342 |
| Iridoviridae | Methanosaeta | 0.393852 | 0.007432 |
| Demerecviridae | Methanosarcina | -0.42322 | 0.003771 |
| Iridoviridae | Methanospirillum | 0.426987 | 0.003442 |
| Ackermannviridae | Methanospirillum | 0.392913 | 0.007588 |
| Autographiviridae | Methanospirillum | 0.386216 | 0.008781 |
| Schitoviridae | Methanospirillum | -0.31491 | 0.035122 |
| Demerecviridae | Myroides | 0.322813 | 0.030554 |
| Ackermannviridae | Nitrosomonas | 0.499061 | 0.000484 |
| Autographiviridae | Nitrosomonas | 0.422065 | 0.003878 |
| Kyanoviridae | Nitrosomonas | 0.357945 | 0.01577 |
| Peduoviridae | Nitrosomonas | 0.316913 | 0.033913 |
| Straboviridae | Nitrosomonas | 0.372581 | 0.011719 |
| Ackermannviridae | Prevotella | 0.423468 | 0.003749 |
| Ackermannviridae | Prevotella 9 | 0.405347 | 0.00574 |
| Iridoviridae | Pseudomonas | -0.29756 | 0.047132 |
| Peduoviridae | Pseudomonas | -0.29536 | 0.048861 |
| Inoviridae | Rhodoferax | 0.303279 | 0.042849 |
| Ackermannviridae | SaccharimonadalesXX | 0.349264 | 0.018693 |
| Autographiviridae | SaccharimonadalesXX | 0.313924 | 0.035727 |
| Straboviridae | SaccharimonadalesXX | 0.32386 | 0.029988 |
| Mimiviridae | Salmonella | -0.30026 | 0.045064 |
| Mimiviridae | Serratia | -0.32909 | 0.027288 |
| Mimiviridae | Streptococcus | -0.34354 | 0.020862 |
| Iridoviridae | WoesearchaealesXX | 0.478774 | 0.000878 |
| Ackermannviridae | WoesearchaealesXX | 0.35548 | 0.016558 |
| Autographiviridae | WoesearchaealesXX | 0.388923 | 0.008281 |
| Schitoviridae | WoesearchaealesXX | -0.4454 | 0.002169 |
| Mimiviridae | uncultured ArdenticatenalesX | -0.44479 | 0.002204 |
| Ackermannviridae | uncultured ArdenticatenalesX | 0.298604 | 0.046323 |
| Autographiviridae | uncultured ArdenticatenalesX | 0.440616 | 0.002452 |
| Kyanoviridae | uncultured ArdenticatenalesX | 0.534744 | 0.000154 |
| Peduoviridae | uncultured ArdenticatenalesX | 0.359616 | 0.015254 |
| Schitoviridae | uncultured ArdenticatenalesX | -0.40257 | 0.006114 |
| Straboviridae | uncultured ArdenticatenalesX | 0.58862 | 2.11E-05 |
| Autographiviridae | uncultured Caldilineaceae | 0.347066 | 0.019502 |
| Straboviridae | uncultured Caldilineaceae | 0.406031 | 0.00565 |
| Autographiviridae | uncultured ChitinophagalesX | -0.31709 | 0.033806 |
| Inoviridae | uncultured ChitinophagalesX | 0.385839 | 0.008853 |
| Peduoviridae | uncultured Comamonadaceae | -0.29695 | 0.047608 |
| Schitoviridae | uncultured Comamonadaceae | 0.320941 | 0.031589 |
| Ackermannviridae | uncultured Saprospiraceae | 0.432529 | 0.003004 |
| Straboviridae | uncultured Saprospiraceae | 0.388844 | 0.008295 |

11. *SEM output*

**Table S8. Model fit indices for the structural equation model (SEM).** Displayed are fit indices used to evaluate the adequacy of the SEM, including chi-square statistics (χ²), degrees of freedom (df), *p*-value, comparative fit index (CFI), non-normed fit index (NNFI, also known as Tucker-Lewis index, TLI), root mean square error of approximation (RMSEA), and standardized root mean square residual (SRMR). Threshold values indicate commonly accepted cutoffs for acceptable or good model fit.

| **Fit index** | **Value** | **Acceptable Threshold Level** |
| --- | --- | --- |
| Chi-square (χ²) | 0.355 | *p* > 0.05 |
| df | 1 | - |
| *p*-value | 0.5513 | > 0.05 |
| CFI | 1 | > 0.95 |
| NNFI (TLI) | 1.204 | > 0.95 |
| RMSEA | 0 | < 0.07 |
| SRMR | 0.0176 | < 0.08 |

**Table S9.** **Output of the structural equation model (SEM) used to assess associations between seasonality and microbial community diversity.** For each tested relationship (Association), the table displays the unstandardized (Estimate) and standardized (Std.Coeff) path coefficients, standard errors (Std.Error), and *p*-values (*p*-value).

| **Association** | **Estimate** | **Std.Error** | ***p*-value** | **Std.Coeff** |
| --- | --- | --- | --- | --- |
| Shannon_Euk ~ Season | -0.12205 | 0.055581 | 0.028101 | -0.32849 |
| Shannon_Vir ~ Season | -0.04557 | 0.01781 | 0.010507 | -0.35638 |
| Shannon_Prok ~ Season | -0.08066 | 0.041633 | 0.052696 | -0.26105 |
| Shannon_Prok ~ Shannon_Vir | 0.871254 | 0.321274 | 0.00669 | 0.360562 |
| Shannon_Prok ~ Shannon_Euk | 0.249605 | 0.10612 | 0.018667 | 0.300143 |
| Shannon_Euk ~ Shannon_Vir | -0.81452 | 0.434667 | 0.060944 | -0.28033 |
| Shannon_ARG ~ Shannon_Vir | 0.110326 | 0.048563 | 0.023097 | 0.352373 |
| Shannon_ARG ~ Shannon_Prok | -0.0509 | 0.020792 | 0.014359 | -0.39284 |
| Shannon_ARG ~ Shannon_Euk | 0.044378 | 0.016027 | 0.005625 | 0.411842 |
